# Actomyosin pulsation and flows in an active elastomer with turnover and network remodeling

**DOI:** 10.1038/s41467-017-01130-1

**Published:** 2017-10-24

**Authors:** Deb Sankar Banerjee, Akankshi Munjal, Thomas Lecuit, Madan Rao

**Affiliations:** 1 Raman Research Institute, Bangalore, 560080 India; 2 IBDM, UMR7288, CNRS-Aix Marseille Universite, Campus de Luminy case 907, 13009 Marseille, France and College de France, Paris, France; 3 Simons Centre for the Study of Living Machines, National Centre for Biological Sciences (TIFR), Bangalore, 560065 India

## Abstract

Tissue remodeling requires cell shape changes associated with pulsation and flow of the actomyosin cytoskeleton. Here we describe the hydrodynamics of actomyosin as a confined active elastomer with turnover of its components. Our treatment is adapted to describe the diversity of contractile dynamical regimes observed in vivo. When myosin-induced contractile stresses are low, the deformations of the active elastomer are *affine* and exhibit spontaneous oscillations, propagating waves, contractile collapse and spatiotemporal chaos. We study the nucleation, growth and coalescence of actomyosin-dense regions that, beyond a threshold, spontaneously move as a spatially localized traveling front. Large myosin-induced contractile stresses lead to nonaffine deformations due to enhanced actin and crosslinker turnover. This results in a transient actin network that is constantly remodeling and naturally accommodates intranetwork flows of the actomyosin-dense regions. We verify many predictions of our study in *Drosophila* embryonic epithelial cells undergoing neighbor exchange during germband extension.

## Abstract (web summary)

Tissue remodeling involves substantial involvement of the contractile actomyosin cytoskeleton. Here the authors model the spatiotemporal evolution of actomyosin densities during *Drosophila* germband extension and find affine and nonaffine deformations that depend on the magnitude of local contractile stress.

## Introduction

Tissue remodeling in diverse developmental contexts such as apical constriction in *Drosophila* ^1–4^, or *Caenorhabditis elegans* ^5^, cell intercalation during *Drosophila* germband extension^6–9^, and xenopus extension and convergence^10^ have been observed to be associated with pulsation and flows of the medial actomyosin cytoskeleton. For instance, tissue extension in the *Drosophila* embryo proceeds by the intercalation of cells, a so-called T_1_-process, initiated by the active shrinkage of a subset of cell junctions called ‘vertical junctions’ (aligned with the dorsal-ventral axis of the embryo, see Supplementary Fig. 1). The junctional shrinkage events are associated with medial-apical actomyosin pulsation and subsequent flow towards this vertical junction^6, 8, 11^. In this paper, we develop a general theory for the dynamics of such spontaneous actomyosin pulsation and symmetry breaking flows, which should be applicable not only in the context of germband elongation, but also during other morphogenetic events. In addition, using germband cells in the *Drosophila* embryo as a model system, we provide experimental justification for the assumptions underlying our theoretical framework and verify many of its key predictions.

**Fig. 1:**
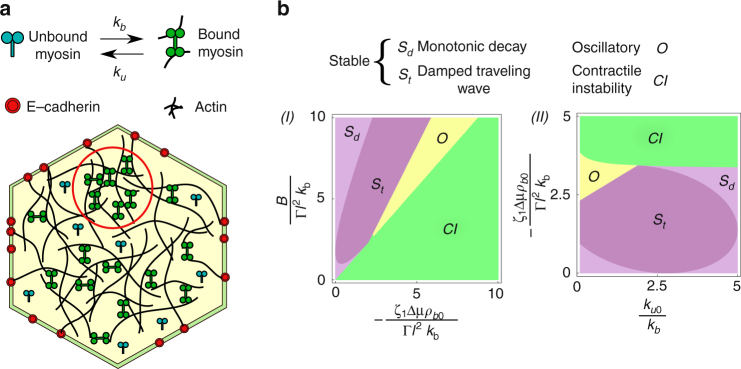
Schematic of apical cortex and linear stability results: **a** Schematic showing the medial actomyosin cytoskeletal meshwork within the apical region of a cell belonging to the tissue. The actin filaments are attached to the cell junctions via E-cadherin (red dots). Myosin minifilaments bind (unbind) with rates *k*_*b*_(*k*_*u*_) and when bound, apply contractile stresses on the actin filament meshwork—the red circle demarcates a region of higher mesh compression. Both actin filaments and myosin minifilaments undergo turnover. **b** (I–II) Linear stability phase diagrams in (I) effective elastic stress density vs. contractile stress density at *k* = 1 and (II) Effective contractile stress density vs. inverse lifetime of bound myosin at *B* = 4. The stresses are normalized by the frictional stress density, Γ*k*_*b*_ *l* ^2^. The phases are described in the legend. Rest of the dimensionless parameters are *α* = 0.1, *c* = 0.1, *D* = 0.1 (see “Methods” section)

The apically located cortical actomyosin cytoskeleton comprises Myosin-II minifilaments, which bind onto a crosslinked actin filament network. The actin mesh is connected to E-cadherin adhesion molecules at the cell junctions via molecular linkers such as α-catenin an actin-binding protein^5, 12–14^ and β-catenin, which binds *α*-catenin and E-cadherin. Here we model the medial actomyosin mesh as an active elastomer embedded in a solvent, subject to active contractile stresses arising from the binding of myosin minifilaments (Fig. 1a)^5, 7, 15, 16^, and turnover of all components. Viewing the spatially resolved time-lapse images and movies (Supplementary Fig. 1 and Supplementary Movie 1) showing the diversity of dynamical regimes, including nucleation, growth, coalescence and flow of clusters of labeled myosin toward the cell junction in a *Drosophila* germband cell, should immediately convince one of the need for adopting a hydrodynamic approach to describe the spatiotemporal evolution of actomyosin densities.

The hydrodynamic equations for this active elastomer are derived from very general arguments based on symmetry considerations and conservation laws, and include minimal phenomenological inputs. When the local contractile stress generated by bound myosin is not too large, the deformations are affine. In this regime, we describe the hydrodynamic modes without inertia and obtain phase diagrams by numerically solving the hydrodynamic equations. Similar equations have been written down in^17, 18^, where the primary focus has been on linear analysis, together with an analysis of the leading nonlinear effects. We find that the active affine elastomer exhibits spontaneous oscillations, contractile instabilities and coarsening of clusters enriched in actomyosin. The coarsening often leads to stable actomyosin-dense clusters that, beyond a threshold, acquire a polarity. This results in spontaneous movement as a spatially localized traveling front. Such localized traveling front solutions appear in other excitable systems such as the FitzHugh-Nagumo model^19^, to which our affine model bears a close resemblance. On the other hand, large myosin-induced contractile stresses can lead to nonaffine deformations due to actin turnover or network rupture. This results in a transient actin network that exhibits large intermittent strain fluctuations and intranetwork flow of the actomyosin-dense regions as a consequence of filament unbinding and rebinding. Interestingly, both the affine and nonaffine theories predict that the driving force for spontaneous movement comes from the actomyosin-dense region itself and not the cell boundary—we provide robust experimental verification of this. Our hydrodynamic analysis of the affine elastomer could be viewed as being closely related to the spring model of^20^, though it goes far beyond this in the nonlinear analysis of the traveling front. Our analysis of the nonaffine elastomer, could be viewed as an active generalization of physical gels^21^ driven by local contractile force dipoles. Our general perspective reveals several significantly new aspects and provides a fresh conceptual understanding of this ubiquitous phenomenon.

## Results

### Active elastomer with turnover and network remodeling

As just stated, we model the medial actomyosin crosslinked mesh as an active elastomer embedded in a solvent, subject to active contractile stresses arising from the binding of myosin minifilaments (Fig. 1a)^5, 7, 15, 16^, and turnover of all components. We will see that this allows us to span the diverse dynamical regimes exhibited by this system. Below, we provide a justification for treating this as an active elastomer with turnover, rather than directly as an active fluid, as was done in the context of *C. elegans* embryo^22, 23^.

We first note that, when the time scales of turnover of the actin mesh or crosslinkers are much smaller than the time scales of macroscopic processes of interest, then the stress relaxation is set by the turnover time scale, and the system should be treated as a fluid. Alternatively, when the actin mesh or crosslinker turnover time scales are much larger than the time scales of macroscopic processes, then the system should be treated as a dissipative elastic medium. The interpretation in these two extreme regimes is unambiguous. However, Fluorescence recovery after photobleaching(FRAP) measurements in germband cells, reveal an actin turnover time of around 10–20 s^6^, which is the same order as the time scale of nucleation and growth, around 10–20 s, and the period of medial actomyosin pulsation, around 50–100 s^6, 8, 11^. Indeed a more detailed look at the movies of myosin (Supplementary Movie 2), show a distribution of time scales and a range of dynamical regimes, starting from the appearance and disappearance of small myosin-rich speckles over a time scale of 5–10 s. This suggests that the appropriate description should span the short-time elastomeric and the longer time fluid-like regimes. As mentioned in the Introduction, we do this by starting out with an elastic description of a mesh, where the local deformations induced by myosin binding and release are affine. Increased myosin binding can lead to rapid turnover of actin and crosslinkers, resulting in loss of network integrity and its fluidization via intranetwork flows; we study this crossover to a nonaffine regime. Indeed, this strategy of going from the short-time elastic to long-time fluidization, as a function of increasing turnover, opens up novel rheological possibilities, such as correlated strain fluctuations and power-law response, which we take up later.

There are several other empirical reasons for starting with an elastomeric description and allowing for turnover of components:

1. Current high-resolution images of medial actin filaments, both in the germband cells and in the amnioserosa, show two distinct populations of actin filaments—a cell-spanning actin filamentous network which appears connected to the cell boundary and a possibly more rapidly turning over pool of shorter actin filaments (T. Lecuit and B. Dehapiot, unpublished observations). The relative levels of these two architectures of actin filaments is likely to be context dependent.
2. The medial actin mesh is connected to the cell junctions; consistent with this, modulating the strength of the coupling of medial actin to the junction via β-catenin affects the pulsation^8^.
3. Finally, the pulsation of medial actomyosin in these systems appears to be correlated with the oscillations in the area of the apical surface^1, 8, 9, 20^.

With this in mind, we shall start our discussion by describing the hydrodynamics of an affine elastomer subject to myosin turnover. We will next explore the consequences of actin turnover and network remodeling, the hydrodynamics of a nonaffine elastomer, giving rise to intranetwork flows and consequent fluid-like behavior.

### Affine elastomer hydrodynamics

The hydrodynamic variables of the confined active elastomer (Fig. 1a) are the actin mesh displacement field *u*, the actin mesh density *ρ*, the density of the bound myosin minifilaments *ρ*_*b*_ (we assume that the unbound myosin constitutes a bath), the junctional E-cadherin density *ρ*_*c*_ and the hydrodynamic velocity *v*. In this section we restrict ourselves to deformations that are affine, i.e., homogeneous deformations over a spatially coarse-grained scale. In the absence of flow and activity, the system relaxes to equilibrium governed by a free-energy functional *F*[***u***,*ρ*,*ρ*_*b*_,*ρ*_*c*_] = *∫*d**r** *f*_B_, where *f*_B_ is the the bulk elastic free-energy density of the elastomer written in terms of the linearized strain tensor ${\it{\epsilon }} = \frac{1}{2}({\nabla {\bf{u}} + {{({\nabla {\bf{u}}} )}^T}} )$ (see “Methods” section). In principle, we need to include a boundary contribution reflecting the soft anchoring arising from the actin-cadherin linkage; for the present, we simply include *ρ*_*c*_’s contribution in the elastic moduli and hard boundary conditions on the dynamical equations.

In the presence of active processes, the so-called Rouse dynamics^24^ without inertia is described by (Supplementary Note 1),

1 $$\Gamma {{\dot u}} = \nabla \cdot \left( {{{\it{\sigma }}^e} + {{\it{\sigma }}^a} + {{\it{\sigma }}^d}} \right)$$

2 $${\dot \rho _b} + \nabla \cdot \left( {{\rho _b}\dot u} \right) = D{\nabla ^2}{\rho _b} + {S_m}$$

3 $$\dot \rho + \nabla \cdot \left( {\rho \dot u} \right) = M{\nabla ^2}\frac{{\delta F}}{{\delta \rho }} + {S_a}$$

Equation (1) is a balance between frictional force experienced by the mesh (with coefficient Γ) and the net force acting on the mesh, written in terms of the total stress ***σ*** ≡ *σ* ^*e*^ + *σ* ^*a*^ + *σ* ^*d*^, a sum of the elastic stress, ${{\it{\sigma }}^e} = \frac{{\delta F}}{{\delta {\it{\epsilon }}}}$, dissipative stress due to the viscosity (*η*) of the elastomer network, ${\sigma ^d} = \eta \nabla \dot u$, and the active stress *σ* ^*a*^. The active stress should depend on the density of myosin and actin - it should increase with increasing myosin density and then saturate - it is thus reasonable to propose that

4 $${{\it{\sigma }}^a} = - \zeta \left( {\rho ,{\rho _b}} \right)\Delta \mu {\kern 1pt} {\it{I}} = - \frac{{{\zeta _1}{\rho _b}}}{{1 + {\zeta _2}{\rho _b}}}\chi \left( \rho \right)\Delta \mu {\kern 1pt} {\it{I}}{\rm{,}}$$

where Δ*μ* is the difference in chemical potential during ATP hydrolysis, *χ*(*ρ*) is some smooth, positive valued function of the mesh density, ***I*** the identity matrix and the overall negative sign, with parameters *ζ*_1_ < 0^25^ and *ζ*_2_ > 0, ensure that the active stress is contractile (this results in a local “negative pressure”, which draws in the surrounding material). Equation (2) describes the dynamics of bound myosin filament density from advection by the filament velocity $\dot u$ and turnover *S*_*m*_ = −*k*_*u*_(*ϵ*)*ρ*_*b*_ + *k*_*b*_ *ρ* by (un)binding. We allow for a possible strain-induced unbinding with a Hill-form, *k*_*u*_(*ϵ*) = *k*_*u*0_ *e* ^***α***⋅***ϵ***^ ^26, 27^. The sign of *α* can be taken to be either positive or negative (Supplementary Fig. 3a): *α* > 0 implies a local extension (compression) of the mesh will increase (decrease) the myosin unbinding, while *α* < 0 implies a local compression (extension) of the mesh will increase (decrease) the myosin unbinding. The choice *α* = 0 implies that the myosin unbinding rate is a constant, independent of mesh deformation. We thus cover all possibilities (for details, see Supplementary Note 3). Similarly, Eq. (3) describes the dynamics of the actin mesh density including advection, permeation (with mobility ***M***) and actin turnover, ***S***_*a*_.

In our analysis, we have ignored the long-range hydrodynamic interactions of the fluid; this is suggested by experiments that show the movement of the actin mesh relative to the fluid does not advect particles suspended in the fluid unless they are bound to the actomyosin mesh^6, 8^. For completeness, however, we display the full hydrodynamic equations in Supplementary Note 1.

From Eqs. (1–3), we note that there is a separation of time scales, which correspond to the advection time scale, myosin turnover time and actin turnover time. The affine description is valid when the actin turnover time is longer than the other times scales. We will consider the case when the actin mesh density is “fast” compared to the time scale associated with the Pec′let number *P*_*e*_ ≡ *L*_*v*_ *V*/*M* (*L*_*v*_ is the characteristic scale on which the mesh velocity, of typical magnitude *V*, varies); in this limit, the mesh density is slaved to the local compressive strain, thus with *ρ* = *ρ*_0_ + *δρ*, we arrive at *δρ* ∝ −*ϵ*_*ii*_, i.e., a local compression of the mesh leads to an increase in actin density (see “Methods” section).

The conceptual features of the affine dynamics are captured by a simple scalar version with one-elastic constant, ***σ*** ^*e*^ = *Bϵ*. It helps to make the equations dimensionless by choosing length and time in units of the screening length $l = \sqrt {\eta /\Gamma }$ and inverse binding rate $k_b^{ - 1}$, respectively; in these units, *u*/*l* → *u*, *ρ*_*b*_/*ρ*_*b*0_ → *ρ*_*b*_, *B*/Γ*k*_*b*_ *l* ^2^ → *B*, *ζ*_1_Δ*μρ*_*b*0_/*k*_*b*_Γ*l* ^2^ → *ζ*_1_Δ*μ*, *D*/*k*_*b*_ *l* ^2^ → *D* and *k*_*u*0_/*k*_*b*_ → *k* are dimensionless. See “Methods” for parameter values.

### Linear analysis

For low levels of bound myosin, the mesh deformation is small. It is thus appropriate to analyze the linear stability about the unstrained, homogeneous steady state (*u* = 0,*ρ*_*b*_ = *ρ*_*b*0_). To this order, the active stress reduces to *σ* ^*a*^ = −*ζ*_1_Δ*μ*(1 + *ζ*′*ρ*)*ρ*_*b*_, with *ζ*_1_ < 0 for contractility and *ζ*′ > 0.

Keeping in mind that −*ζ*_1_Δ*μ*, *B*, and *D* are the dimensionless active stress, elastic modulus and myosin diffusion, respectively, our linear stability analysis demonstrates that: (i) when the active stress is smaller than the elastic stiffness, $- {\zeta _1}\Delta \mu < (B + D{(1 + \sqrt {k/D} )^2})/2$, the elastic mesh is stable with dispersion $\omega \sim {q^2}$, (ii) as the contractile strength exceeds $( B + D{(1 + \sqrt {k/D} )^2})/2$, the elastomer undergoes unstable oscillations with an amplitude that increases exponentially with time, (iii) at a threshold boundary, the elastomer supports a traveling wave solution with a speed $v_c^* = \root 4 \of {{{( {kD{B^2}/4} )}}}$, (iv) beyond an active stress $- {\zeta _1}\Delta \mu \sim B$, the elastomer contracts indefinitely.The phase diagrams shown in Fig. 1b reflect these four phases. Since this kind of linear analysis appears in^17, 18^, we simply state our results here and refer to Supplementary Note 2 and Supplementary Fig. 2 for details of calculations and dispersion relations.

In addition, we find that the qualitative features of these transitions remain unaltered when the strain-dependent unbinding parameter *α* is varied, as long as *α* ≤ *α*_max_(*B*,*ζ*_1_Δ*μ*). This is discussed in detail in Supplementary Note 3 and Supplementary Fig. 3.

In spite of the simplifying nature of the linear analysis, it captures some gross features and offers some useful hints: (a) although unstable, the oscillatory behavior or pulsation (which will be stabilized by nonlinearities) requires advection and myosin turnover^8, 28^, (b) the contractile instability is promoted by lowering elastic stiffness (by reducing levels of β-catenin/cadherin)^5, 7, 15^ or reducing myosin unbinding rates^8, 28, 29^. The linear analysis, however, fails to capture finite-amplitude sustained oscillations and the phenomenology of moving configurations of actomyosin.

### Leading order nonlinearities

It is possible that the oscillatory instability obtained in the linear analysis is tempered by the nonlinear terms in the dynamics, arising from strain-dependent unbinding *k*_*u*_(*ϵ*), advection and active stress. Indeed, we find that by taking into account the nonlinear contributions to leading order, we can recast the equations as an excitatory-inhibitory dynamical system that exhibit sustained spontaneous oscillations. To see this, we work in 1-dim, and after fourier transforming Eqs. (1),(2) in a finite domain [0, *L*] with Neumann boundary conditions, retain only the smallest wavenumber, a 1-mode Galerkin truncation, which accomodates the strain-dependent unbinding nonlinearity. The resulting coupled ODEs upon re-scaling describe a generalized van der Pol oscillator^30^ with linear damping and cubic nonlinearities. This admits a limit cycle through a supercritical-Hopf bifurcation for $- {\zeta _1}\Delta \mu \ge \frac{B}{2} + \frac{1}{{{\pi ^2}}}$—a signature of the appearance of sustained spontaneous oscillations. At the onset of bifurcation, we use a fluctuation analysis to obtain the time period of oscillation *T* = 2*π*(*η*/*k*_*u*0_(*B* + *ζ*_1_Δ*μρ*_*b*0_))^1/2^. To see the effects of the advective nonlinearity, we need to extended the above mode-truncation analysis to 2-modes. The resulting 3-dimensional dynamical system exhibits, in addition to limit cycles, temporal chaos as seen by the algebraic decay of the power-spectrum, positive Lyapunov exponent and denseness of the Poincare section (D.B., manuscript in preparation). However, our attempt to include nonlinear effects using low order mode-truncation, fails to capture the phenomenology of moving configurations of actomyosin.

Note that wave-like dispersion relations obtained from this truncated model are not a response to an external perturbation, but are self-generated. This cell-autonomous behavior is consistent with observations of pulsatile dynamics in medial actomyosin^6, 11^.

### Full nonlinear analysis shows traveling front solutions

We now study the full nonlinear theory, which includes the effect of the nonlinearity arising from the active stress. This gives rise to a new set of solutions, namely the spatially localized traveling front solutions. To see this, we perform a numerical analysis of the full nonlinear equations in 1-dim. We first Taylor expand *χ*(*ρ*) in Eq. (4) about *ρ*_0_, the mesh density in the unstrained configuration, and recast the active stress as (see “Methods” section)

5 $${\sigma ^a} = \frac{{ - {\zeta _1}\Delta \mu {\rho _b}}}{{1 + {\zeta _2}{\rho _b}}}\left( {\chi \left( {{\rho _0}} \right) - c{\chi ^{\prime}}\left( {{\rho _0}} \right)\epsilon + {c^2}{\chi ^{\prime\prime}}\left( {{\rho _0}} \right){\epsilon ^2} + \ldots } \right),$$

where *c* is a positive constant (see “Methods” section). Separating out the terms dependent on *ϵ* and only on *ρ*_*b*_, and combining the former with the elastic stress *σ* ^*e*^ = *Bϵ* in Eq. 1, leads to an effective “elastic free-energy”,

6 $${\rm{\Phi}} \left( \epsilon \right) = \frac{1}{2}{K_2}\left( {{\rho _b},{\rho _0}} \right){\epsilon ^2} + \frac{1}{3}{K_3}\left( {{\rho _b},{\rho _0}} \right){\epsilon ^3} + \frac{1}{4}{K_4}\left( {{\rho _b},{\rho _0}} \right){\epsilon ^4},$$

where *K*_*i*_ (*i* = 1, 2, 3) are density dependent coefficients (see “Methods” section), and the quartic term with *K*_4_ > 0 ensures that the local compressive strain does not grow without bound, as a consequence of steric hinderance, filament rigidity or crosslinking myosin. The Φ(*ϵ*) that emerges as a consequence of activity, has 3 new features: (i) for weak active contractile stress, the minima at *ϵ* = 0 gets shallower, indicating that the elastic stiffness *B* decreases, (ii) as we increase the active stress, there appears another minimum at *ϵ* = *ϵ*_0_ (iii) for large active stresses, the *ϵ* = 0 state can be unstable, with the effective *B* < 0 (Supplementary Fig. 4). The final 1-dim equations of motion are given by,

7 $${\rm{\Gamma}} \dot u = {\partial _x}{\Phi ^{\prime}}\left( \epsilon \right) + {\partial _x}{\sigma ^a}\left( {{\rho _b}} \right)$$

8 $${\dot \rho _b} = - {\partial _x}\left( {{\rho _b}\dot u} \right) + D\partial _x^2{\rho _b} + {{S}_m}\left( {\epsilon ,{\rho _b}} \right),$$

where ${\Phi ^\prime } \equiv \frac{{\delta \Phi }}{{\delta \varepsilon }}$, ${\sigma ^a}({\rho _b}) = \frac{{ - {\zeta _1}{\rm{\Delta }}\mu {\rho _b}}}{{1 + {\zeta _2}{\rho _b}}}\chi ({\rho _0})$ and myosin turnover ***S***_*m*_(*ϵ*,*ρ*_*b*_) = −*k*_*u*0_ *e* ^*αϵ*^ *ρ*_*b*_ + *k*_*b*_(1 − *cϵ*).

**Fig. 2:**
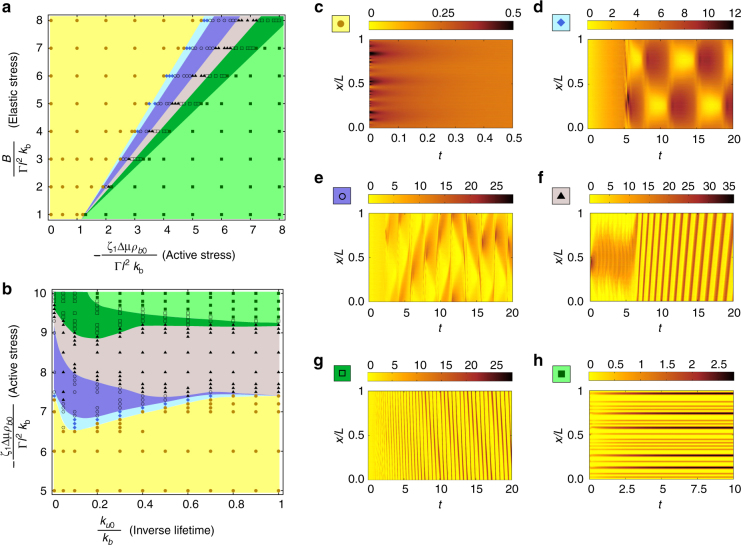
Phase diagram obtained from numerical solutions of Eqs. 7 and 8. **a** Effective elastic stress density vs. contractile stress density, with *k* = 0.1. **b** Effective contractile stress density vs. inverse-lifetime, with *B* = 5. The phases are (i) Stable (yellow), (ii) spontaneous Oscillatory (blue), (iii) spontaneous Moving (grey) and (iv) contractile Collapse (light-green). The corresponding kymographs of the bound myosin density (indicated by the color and symbol on the upper right corner) is shown in **c**, **d**, **f** and **h**, respectively. The regions marked violet and dark-green are the coexistence phases—the oscillatory-moving coexistence (open circle) and the collapse-moving coexistence (open square), with the corresponding kymographs shown in **e** and **g**, respectively. Apart from the new phases, the topology of the phase diagrams are roughly similar to the linear stability diagram (Fig. 1b), except for the upturn of the phase boundaries towards larger active stress in **b**, which arises from the nonlinear strain-dependent unbinding. Symbols are points at which numerical solutions have been obtained. Rest of the dimensionless parameters are, *α* = 3, *c* = 0.1, *χ*(*ρ*_0_)*ζ*_1_ = −0.5, and *ζ*_2_ = 0.1 (see “Methods” section)

These equations are numerically solved with either periodic or Neumann boundary conditions using a finite difference scheme (see “Methods” section). Initial conditions are small amplitude random fluctuations about the homogeneous unstrained state. The numerical phase diagram, displayed in Fig. 2 shows several new features compared to the linear phase diagram, which we discuss below.

### Steady-state phase diagram

The two features that are expected to arise from nonlinear effects, namely, the tempering of the linear instabilities to obtain both finite-amplitude oscillatory and finite-amplitude contractile collapse phases at intermediate and high contractile stresses, respectively, show up in the steady-state phase diagram, Fig. 2a, b. The corresponding kymographs in the bound myosin density (Fig. 2d, h) show the appearance of these steady state at late times. The time development of configurations in these phases can be summarized as follows—starting from a generic state with small random fluctuations about the homogeneous unstrained state, the configuration quickly results in a spatially heterogenous (un)binding of myosin filaments onto the actin mesh, transiently generating localized compression. This will increase the local concentration of actin, which in turn will facilitate more myosin recruitment and hence more compression. This local compression will be resisted by an elastic restoring force, and the resulting strain can lead to an enhanced myosin unbinding. If it does, this will lead to a relaxation of the compressed region, to be followed by another round of binding-compression-unbinding, leading to the observed oscillations. In this spontaneous oscillating phase, the frequency gets smaller with increasing the active stress or decreasing unbinding rate^8^. On the other hand, if myosin unbinding does not occur fast enough, the elastomer will undergo a contractile instability, to be eventually stabilized by nonlinear effects such as steric hinderance and filament rigidity.

Additionally, there is a wholly unexpected feature that emerges from a numerical solution of the full nonlinear equations. In the parameter regime between the oscillatory and the contractile collapse phases, there appears a moving phase (Fig. 2), where spatially localized actomyosin-dense regions (which we later identify as traveling fronts) spontaneously move to either the left or right boundary. In the regimes between the pure moving phase and the oscillatory and collapse phases lie the coexistence phases where the moving phase coexists with oscillations and collapse, respectively. The corresponding kymographs in the bound myosin density (Fig. 2e–g) show the appearance of these steady state at late times. We may understand the occurrence of these phase transitions using a simple argument based on the relative time scales of these dynamical events, as displayed in Supplementary Fig. 5.

Several qualitative assertions follow immediately from the affine theory, such as: (i) the existence of bounded (finite-amplitude) oscillations requires both strain-dependent unbinding and turnover of myosin, (ii) the coexisting oscillation-moving and collapse-moving phases cannot be obtained in the absence of strain-dependent unbinding, (iii) advection is a necessary condition for front movement. We now look more closely at the dynamics of actomyosin-dense regions and compare the results of the affine theory with early time dynamics of myosin dense regions in vivo.

### Nucleation, growth and coalescence

**Fig. 3:**
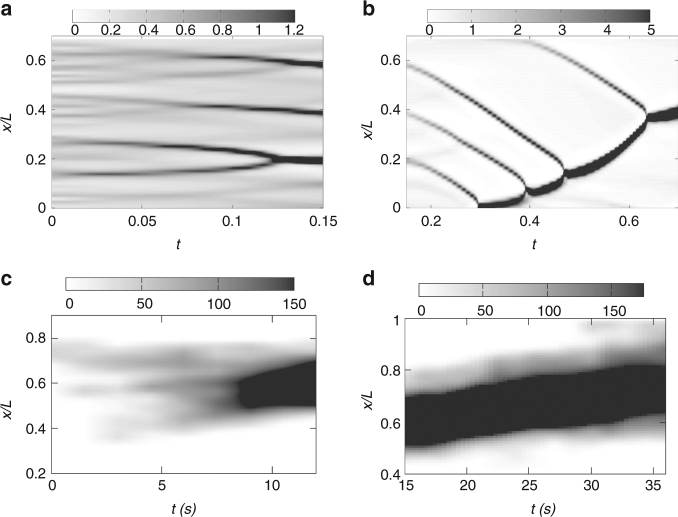
Kymographs of bound myosin density from theory and experiment: **a**, **b** Kymograph of the spatial profile of bound myosin density from theory, shows nucleation and growth (0 < *t* < 0.15), followed by coalescence (0.15 < *t* < 0.7) and movement (*t* > 0.7). Here *B* = 6, −*ζ*_1_Δ*μ* = 5.5, *k* = 0.5, *α* = 1 and *D* = 0.15. Rest of the parameters as in Fig. 2. **c** Kymograph of the spatial profile of labeled myosin from experiment, shows nucleation and growth (0 < *t* < 5 s), followed by coalescence (5 < *t* < 10 s) and **d** eventual movement of the formed actomyosin-rich region (*t* > 10 s)

In the moving regime, the effective ‘elastic free-energy’ functional Φ(*ϵ*) develops a second minima at *ϵ* = *ϵ*_0_ corresponding to a local compression due to contractility (Supplementary Fig. 4). Initiation of movement starts with the nucleation of actomyosin-dense regions, which grow and coalesce to form larger actomyosin-dense regions. This is best seen using a space–time analysis of Eqs. 7–8 with initial conditions stated above. Kymographs of the spatial profile of bound myosin density, calculated from the theory show nucleation and growth (0 < *t* < 0.15), followed by coalescence (0.15 < *t* < 0.7) and eventual movement (*t* > 0.7) (Fig. 3a, b). This space–time behavior accurately recapitulates the early time dynamics of medial myosin in vivo as seen from the experimental kymographs, Fig. 3c, d.

### Asymmetric actomyosin profile of traveling front

**Fig. 4:**
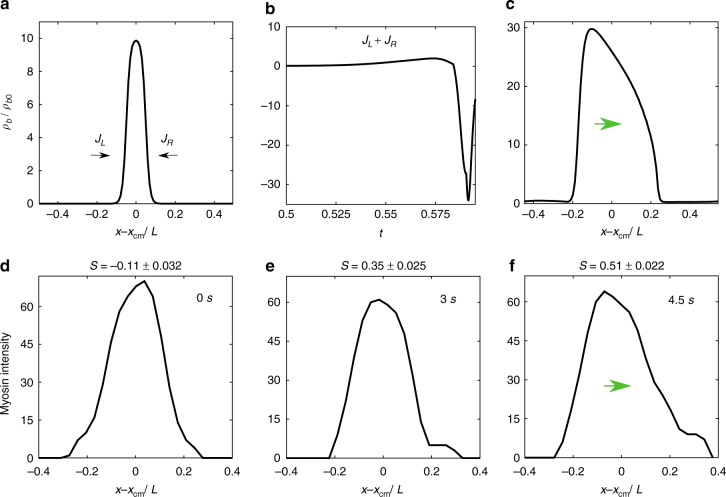
Time evolution of *ρ*_*b*_, the density profile of bound myosin in a cluster prior to movement: **a** Prior to movement the myosin density profile is seen to be symmetric, following which we compute the instantaneous left-right fluxes, *J*_L,R_ of myosin drawn into it. **b** Time evolution of the algebraic sum *J*_L_ + *J*_R_, shows that after a while, there develops a net flux in one direction as a precursor to the asymmetric traveling front. **c** Emergence of the asymmetric traveling front which moves towards the right in a shape-invariant manner. **d**–**f** Myosin intensity profiles from experiments, which shows how an initial stationary symmetric myosin profile at *t* = 0, finally evolves to an asymmetric profile at *t* = 4.5 s, which then travels to the right. The degree of shape asymmetry of the profiles is described by the skewness *S*, the standard error of mean(s.e.m.) reported is due to projection of the images to one dimension

Here we investigate the origins of the spontaneous movement of the actomyosin-dense region. We study the configuration of the localized actomyosin-dense region just prior to movement, and find that it assumes a symmetric localized profile (Fig. 4a) within which the strain *ϵ* = *ϵ*_0_ (the second minimum) where Φ′(*ϵ*) = 0. The active stress within this region is higher than outside, the resulting gradient in stress should induce inflowing myosin currents from either side of it. We verify this by monitoring the fluxes *J*_*L*_ and *J*_*R*_, coming from the left and right of this symmetric profile. Over time, owing to stochasticity either in the initial conditions or the dynamics, there is a net flux, *J*_*L*_ + *J*_*R*_, from either the left or the right (Fig. 4b), leading an asymmetric profile (Fig. 4c), and hence a gradient of *ρ*_*b*_ across the profile. This marks the onset of the traveling front. This feature also appears to be present in the early time dynamics of the moving myosin profiles observed in vivo, as seen in Fig. 4d–f.

**Fig. 5:**
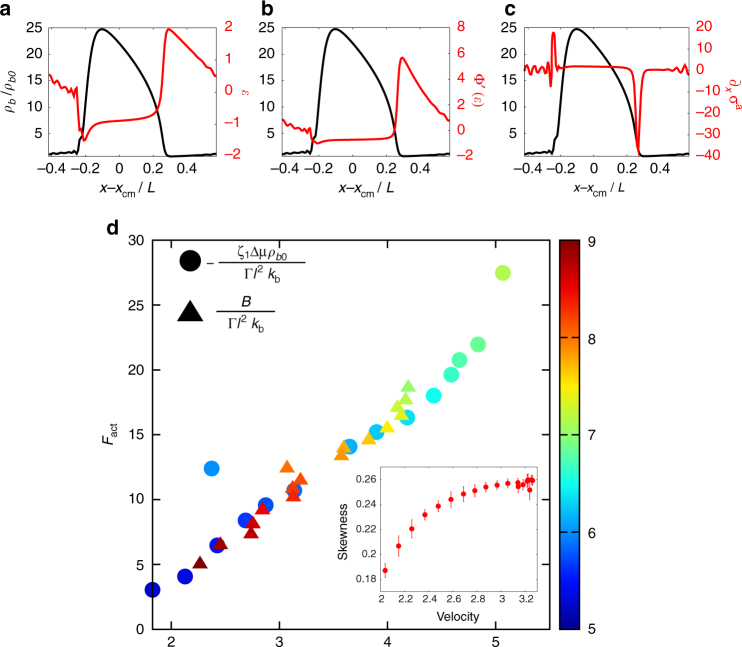
Anatomy of the traveling front in the co-moving frame: **a** Spatial profile of excess bound-myosin density (black) and strain *ϵ* (red) profile, **b** Spatial profile of Myosin density and derivative of the effective elastic free energy Φ′(*ϵ*) (red), **c** Spatial profile of myosin density and active force (red). Horizontal axis is distance from centre of mass position, *x*_CM_. Here *B* = 6.0, −*ζ*_1_Δ*μ* = 4.8, *k* = 0.2, *α* = 1.0 and *D* = 0.25. **d** Theory predicts that the traveling front velocity is proportional to the net active force integrated over the front profile across the moving front. We demonstrate this fact from a numerical solution of the dynamical equations by varying the parameters of the active stress (circle) and the elastic stress (triangle). for different values of *B* (with −*ζ*_1_Δ*μ* = 6.0, *k* = 0.2 and *D* = 0.15 fixed) and −*ζ*_1_Δ*μ* (with *B* = 8.0, *k* = 0.2 and *D* = 0.15 fixed). Rest of the parameters as in Fig. 2.The color bar shows the magnitude of these stresses in dimensionless units. (inset) Skewness of bound myosin profile in the traveling front vs. velocity, obtained by varying the contractile stress −*ζ*_1_Δ*μ* from 3–4 shows a linear increase followed by saturation. The other dimensionless parameters are: *B* = 4, *k* = 0.2, *D* = 0.1, *α* = 1 and *c* = 0.1. The error bars are calculated as s.e.m

The affine theory predicts that the myosin density profile is asymmetric and moves as a traveling front, with a constant velocity while maintaining its shape (as long as there are no further coalescence events). We confirm this using a variety of initial conditions of the bound myosin density *ρ*_*b*_, including starting with a single symmetric gaussian profile. We analyze the asymmetric profile of the traveling front by transforming to the co-moving frame *x* ± *vt* (Fig. 5).

We find that within the traveling front, the strain takes a value slightly more compressed relative to *ϵ*_0_, the value of the strain at the second minima, where Φ′ = 0. The traveling front is stably compressed in a force-free state (Fig. 5a, b). The asymmetric myosin profile gives rise to a gradient in the active stress (Fig. 5c), which provides the propulsion force for the traveling front to move to the right in Fig. 5c.

### Dynamics of traveling front

The dynamics of the traveling front that emerges from the affine theory is local, its propulsion is therefore independent of the boundary or the distance from the boundary. We calculate the velocity of the traveling front by integrating Eqs. (7 and 8) across the scale Ω of the traveling front in the co-moving frame. This leads to the formula, *v* = Γ^−1^ *∫*_Ω_∂_*x*_ *σ* ^*a*^ ≡ Γ^−1^ *f*_*act*_, which states that the velocity depends only on the shape asymmetry of the front; if the shape is maintained over time, then the velocity is a constant. In Fig. 5d, we plot the *f*_*act*_ vs. the velocity and show that they are proportional to each other over a large range of active and elastic stresses. We will later make connection with experiments, where it is convenient to use the shape asymmetry or skewness of the myosin profile, $S \equiv \frac{{{\int}_{\rm{\Omega }} {{( {x - {x_{CM}}} )}^3}{\rho _b}(x)}}{{{{( {{\int}_{\rm{\Omega }} {{( {x - {x_{CM}}} )}^2}{\rho _b}(x)} )}^{3/2}}}}$, as a proxy for the driving force. We find that the traveling front velocity increases with increasing skewness before saturating at larger velocities (inset of Fig. 5d).

### Affine theory predicts moving deformation

**Fig. 6:**
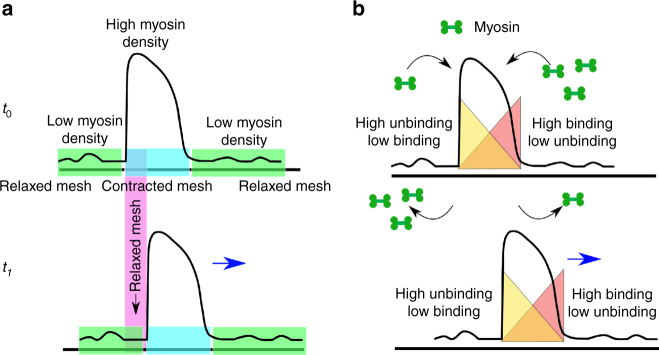
Possible mechanisms for the movement of actomyosin-dense structures in an active affine elastomer: **a** moving deformation of the actomyosin mesh without turnover, implying a traveling front, and **b** moving deformation of the actomyosin mesh with differential myosin binding unbind rates at the leading and trailing edges of the front

It is important to note that the movement of the actomyosin-dense region arising from affine deformations of the active elastomer is a moving deformation of the actomyosin mesh, and once established, is not contingent on myosin turnover, as shown in Fig. 6a. One could also sustain a traveling front or moving deformation of the actomyosin mesh by ensuring a differential myosin binding and unbinding rates at the leading and trailing edges of the front, in a kind of treadmilling movement, Fig. 6b. None of these however is associated with mass flow of actin and myosin. Indeed, the dynamical equations describing the active affine elastomer, Eqs. 7–8, bear a close resemblance to the generalized FitzHugh-Nagumo model, an excitable system which is known to exhibit traveling front solutions^19^.

### Nonaffine elastomer with network remodeling and intranetwork flow

**Fig. 7:**
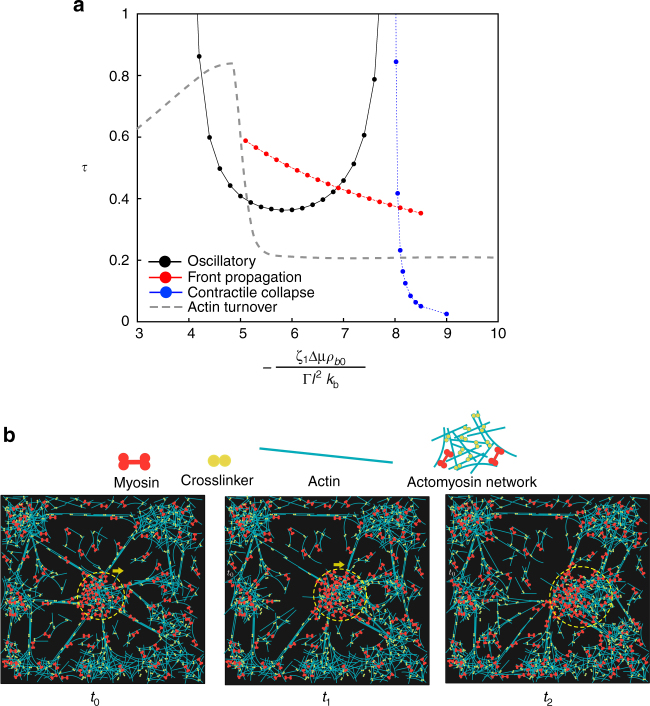
Enhanced actin turnover results in nonaffine deformation of the elastomer: **a** Qualitative behavior of the actin turnover time as a function of contractile stress, which is consistent the with data from refs. ^31–33^. This should be compared with the time scales of oscillation, front propagation and contractile collapse obtained from the affine theory (see, Supplementary Fig. 5). Based on the discussion in the text, we have placed the crossover stress ${\sigma ^ \star }$ in the moving regime, thus implying that the crossover to the nonaffine description occurs in this regime. **b** Schematic showing the intranetwork flow of an actomyosin-dense region (enclosed within the yellow circle) resulting from active stress induced unbinding and rapid turnover of the actin in a transient actomyosin network

As time progresses and the myosin-dense clusters grow, the local contractile stresses can become large. Such large contractile stresses may dramatically enhance actin turnover and crosslinker unbinding. In Fig. 7a, we give a schematic plot of the actin turnover time as a function of contractile stress, and compare it with the time scales of oscillation, front propagation and contractile collapse obtained from the affine theory. Indeed there is experimental evidence that actin turnover times at first increase with increasing contractility^31–33^ - this is likely due to the fact that bound myosin might occupy the binding sites of actin remodeling proteins such as cofilin. There is also evidence that for large levels of contractility, the actin turnover rates are large, possibly because of destabilization of actin or unbinding of crosslinkers at large contractile stress. We make the plausible assumption that the qualitative change in turnover rates is sudden at a stress scale ${\sigma ^ \star }$ (Fig. 7a), beyond which the deformation of the mesh can no longer be considered affine.

We use this insight to arrive at a nonaffine description of the active elastomer. Consider a disordered mesh comprising actin filaments linked to each other by crosslinkers such as α-actinin and myosin (we will assume that this is an unentangled network). The bound myosin locally compresses the mesh here and there, recruiting more myosin in the process. When the local bound-myosin concentration goes beyond a threshold (so that the configuration now samples the second minimum of the effective free energy, Φ(*ϵ*)), the local compression is high and the mesh surrounding this myosin-dense region gets significantly stretched. This could lead to a tearing or ripping of the mesh, either via the unbinding of crosslinkers, destabilization of actin or by the sliding and slipping of filaments past each other. This mesh breakage subsequently heals by the rebinding of crosslinkers or actin itself.

With this picture in mind, we refer to several seminal studies on thermally activated reversibly crosslinked networks in the context of the dynamic properties of physical gels^21, 34, 35^. The most dramatic feature of such reversible networks is its internal fluidity, where each chain can diffuse through the entire network due to the finiteness of the crosslinker life time, in spite of being partially connected to the macroscopic network structure in the course of movement. These systems thus flow under an external stress on time scales longer than the crosslinker dissociation time.

In the context of actomyosin networks, the disrupting influence of filament turnover and the ultimate fluidization has been the subject of some study^36^. In a very recent submission, the healing effect of turnover has also been investigated, and long-range network flows have been demonstrated in simulations of a model actomyosin cortex^37^.

In the present context, when the local bound myosin concentration rises beyond a threshold and attains an asymmetric density profile, it induces mesh breakage in its surrounding regions. This actomyosin-dense region can move through the entire network due to the finiteness of the crosslinker life time, in spite of being partially connected to the macroscopic network structure in the course of movement. These systems should thus exhibit flow under an internal active contractile stresses on time scales longer than the crosslinker dissociation time. This is depicted in Fig. 7b.

To describe this mathematically, it is convenient to define physical quantities coarse-grained over the scale of the actomyosin-dense region Ω, such as ${\bar {\rho} _b} = {{\rm{\Omega }} ^{ - 1}}{\int}_{\rm{\Omega }} {\rho _b}$ and **p** = Ω^−1^ *∫*_Ω_∇ ⋅ ***σ*** ^*a*^, the net force-dipole associated with the anisotropy of the myosin profile. We may decompose myosin density configuration ${\bar \rho _b}$ into a sum of actomyosin-dense clumps (contributing to nonaffine deformations) with volume fraction *ϕ* and a background (contributing to affine deformations). The equation for the ${\bar \rho _b}$ may now be written as,

9 $${\overline \rho _b} = - \nabla \cdot \left( {{{\overline \rho }_b}{v}} \right) + D\left( {1 - \phi } \right){\nabla ^2}{\overline \rho _b} + {S_m}\left( {{{\overline \rho }_b}} \right),$$

where, *v* = *ϕβ*(**p** + *γ* **p**⋅*ϵ*) + (1 − *ϕ*)${\dot u}$, and *β* is the mesh breakage probability and *γ* is a strain-alignment parameter.

Flow described here is a consequence of internal active deformations in a transient actomyosin network. We believe this is a new physical phenomenon; unlike the flow observed in physical gels under external load, this active flow is generated by internally generated stresses even in the absence of an external load.

In general, the interplay between actively generated contractile stresses and stress-dependent turnover of components that shows up in many cellular contexts, promises a rich phenomenology with novel rheological consequences. The constant remodeling and turnover of the actin mesh could drive the system from an elastic regime to a fluid-like behavior via a critical elastic state, which is characterized by correlated strain fluctuations, which might be intermittent. A more complete hydrodynamic theory of nonaffine deformations of a actomyosin network with turnover is a task for the future.

### Comparison with experiments

In making comparisons of the theory presented here with experiments in germband cells in vivo, it is important to demarcate the affine and nonaffine regimes of the elastomer. The difficulty in doing this, is that, we do not know the detailed physical mechanism that would allow us to compute the form of the actin turnover time as a function of contractile stress (Fig. 7a). Nevertheless, it is clear that starting from an unstrained elastomer, the early time dynamics should be described by the linear and leading nonlinear analysis of the affine theory, as described earlier. The dynamical behaviors described by this analysis include oscillations and contractile collapse, from which we make the following qualitative assertions: (i) the existence of bounded (finite-amplitude) oscillations requires advection, strain-dependent unbinding and turnover of myosin, (ii) the coexisting oscillation-moving and collapse-moving phases cannot be obtained in the absence of strain-dependent unbinding. As the kymographs in Fig. 2 show, these dynamical behaviors start emerging at *t* ≈ 2 − which translates to a real time of ≈6 s (see “Methods” section), which is smaller than the actin turnover time of around 10–20 s^6^.

The stable, oscillatory and the collapse phases of the affine theory compare favorably with in vivo experiments in germband cell^8^. First, our phase diagram showing the stable, oscillatory and contractile collapse phase is grossly consistent with the experimental phase diagram that appears in ref. ^8^. Pulsatory solutions are obtained over a wide range of parameters, which include the pure oscillatory and the coexistence phases (Fig. 2d–g). In addition, we compare the finer aspects of the oscillatory phase with the pulsation seen in experiments (Supplementary Fig. 10). Consistent with ref. ^8^, we see that advection is crucial to obtain oscillations of bound myosin, both locally (Supplementary Fig. 10a, b) and cell-averaged (Supplementary Fig. 10(c)). We find a strong correlation between convergent (divergent) advection velocities and increased (decreased) myosin density (Supplementary Fig. 10a–c). Moreover, the amplitude of the oscillation decreases when we reduce actin density *ρ*_0_ (Supplementary Fig. 10d), which is consistent with the actin perturbation experiments in ref. ^8^.

Now the nucleation, growth, and coalescence events of the myosin-dense clusters, precursors to the eventual moving phase of the affine theory, also take place over these time scales and compare well with the in vivo kymographs (Fig. 3). The establishment of the asymmetric profile of the myosin-dense cluster at the onset of movement also appears to be present in the early time dynamics of the moving myosin profiles observed in vivo, Fig. 4d–f.

The moving (traveling front) solution is the one significant prediction of the full nonlinear affine theory, and appears at later times. By this stage, the local contractile stresses are high, opening up the possibility of a crossover to the nonaffine regime. Both the affine and nonaffine theories predict movement—the affine theory predicts a moving deformation or a traveling front, while the movement in the nonaffine theory is associated with mass flow as a consequence of the steady turnover of actin filaments.

To test which of these pictures is true in vivo, we appeal to FRAP experiments performed on a small region within the actomyosin-dense cluster^6^. These preliminary experiments show loss of recovery upon FRAP, suggesting the possibility of actual mass flow, consistent with the predictions of the nonaffine theory (see Supplementary Information in ref. ^6^).

**Fig. 8:**
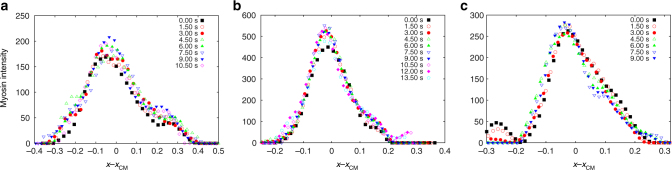
Spatial profile and movement of myosin dense regions: Excess bound myosin density in the moving frame. **a**–**c** Three separate examples of the spatial profile of myosin intensity in the co-moving frame of the flowing actomyosin-dense cluster displayed at different times. Note that the actomyosin-dense cluster hardly changes its shape as it flows towards a junction

The fact that the actomyosin-dense region commences to move with a constant velocity when it has attained an asymmetric profile, is common to both the affine and nonaffine theory. It moves with a constant velocity in the direction where the leading edge has the smoother slope and maintains its asymmetric shape as it moves. This appears to be consistent with the situation in vivo, Fig. 8a–c.

**Fig. 9:**
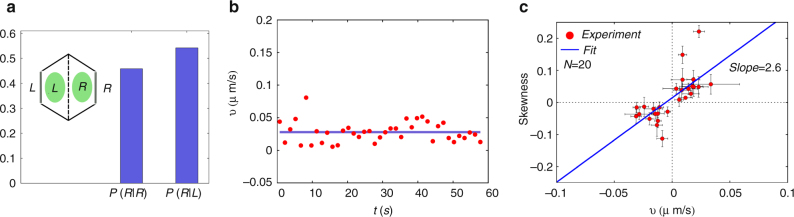
Velocity and profile of myosin dense regions: **a** Histogram of the number of flows that move to the right junction *R* starting from either the right *R* (*P*(*R*|*R*) or the left *L* (*P*(*R*|*L*) region of the cell (inset shows schematic). The data collected from 24 actomyosin-dense clusters over 18 cells. The fact that the histograms are similar is consistent with the theoretical prediction that the flow is spontaneous and not driven by the cell boundaries. **b** Velocity of an isolated flowing actomyosin-dense cluster monitored over time shows that it is a constant, as predicted by theory. **c** Here we present average skewness values of 28 different pulses plotted against average velocities of the respective pulses. The linear behavior is quite clear from the data and a linear fit produced a slope value of 2.6 ± 0.5. This intensity data set was prepared from 20 myosin-GFP-tagged germband cells (*N* = 20). Error bars indicate s.e.m. of fluctuation of skewness and velocity values of a pulse in time

The driving force for this movement is established within the medial actomyosin-dense cluster and not the cell boundary. Its propulsion is therefore independent of the boundary or the distance from the boundary. We ask whether this is true of the moving actomyosin-dense regions in vivo. Figure 9a shows that irrespective of its initial position at the commencement of the flow, the moving actomyosin-dense region travels to the left or right cell boundary with equal probability. Further, Fig. 9b shows that the moving actomyosin-dense region travels with a constant velocity as it moves towards a given cell boundary, its speed does not depend on the distance from the cell boundary.

From the myosin intensity, we compute the shape asymmetry via the skewness, $S \equiv \frac{{{\int}_{\rm{\Omega }} {{( {x - {x_{{\rm{CM}}}}} )}^3}{\rho _b}(x)}}{{{{( {{\int}_{\rm{\Omega }} {{( {x - {x_{{\rm{CM}}}}} )}^2}{\rho _b}(x)} )}^{3/2}}}}$, of the myosin profile, and find that it is proportional to the speed of the moving myosin-dense cluster (Fig. 9c).

To our mind, this establishes unambiguously that the flow toward the junctions is spontaneous with the driving force coming from the gradient in myosin established within the front. The boundary does not affect the flow speed, at best, weak asymmetries that may arise at the boundary (for instance due to an asymmetry in functional cadherin) may bias the direction of the flow^7^.

One consequence of this is that a cluster moving to the right, might reverse its direction following a coalescence with another moving cluster; such reversals are observed in in vivo experiments (Supplementary Fig. 7 and Supplementary Fig. 9).

## Discussion

Although simplified, in that it has completely ignored the coupling of actomyosin dynamics to local chemical signaling such as Rho^8^, we believe this active elastomer model with strain-dependent turnover of components, admitting both affine and nonaffine deformations, captures the essential physics of actomyosin pulsation and flows observed in a wide variety of tissue remodeling contexts such as *Drosophila* germband extension and dorsal closure in the amnioserosa. The minimal ingredients for actomyosin pulsation and flow are mesh-elasticity, actomyosin contractility, advection and turnover of both myosin and actin.

In this study, we have modeled the medial actin mesh during apical constriction and germband extension in the *Drosophila* embryo as an active elastomer embedded in a solvent^5, 7, 15, 16^, which undergoes turnover of all its components. Our description goes from the hydrodynamics of an affine elastomer to a nonaffine elastomer, the latter incorporating network rupture and remodeling, resulting in intranetwork flows. It thus goes from an elastomer to a fluid-like description. Together, the affine and nonaffine active elastomer model captures the range of dynamical regimes exhibited in this system.

To make a detailed comparison of the spatiotemporal actomyosin patterns with experiments generated using quantitative imaging, we will need to extend this numerical study to 2-dimensions, using appropriate (anisotropic) boundary conditions and allowing for shear. The nucleation and growth of the actomyosin-rich domains are similar to that seen in 1-dim, with the difference being that domains can move around each other in 2-dim and can exhibit anisotropic movement.

In future, we would like to extend this framework to understand the dynamical coupling of the medial actomyosin with degrees of freedom (concentration of E-cadherin) attached to a deformable cell junction. Though the emergence of actomyosin flows does not depend on specific boundary conditions, cell boundaries may directionally bias the intrinsic ability of actomyosin networks to generate flow, as proposed before^7^.

## Methods

### Free-energy functional

The bulk elastic free-energy density of the elastomer in terms of the linearized strain tensor *ϵ*_*ij*_ is given by ${f_B} = \frac{\mu }{2}{\epsilon _{ij}}{\epsilon _{ij}} + \frac{\lambda }{2}{\epsilon _{ii}}{\epsilon _{jj}} + \ldots + C\delta \rho {\epsilon _{ii}} + \frac{A}{2}\delta {\rho ^2} + \ldots$, where the … represent higher-order terms to stabilize a possible activity induced contracted state and to prevent a runway density increase (*δρ* is the deviation of the local mesh density from its equilibrium value, *δρ* = −(*C*/*A*)*ϵ*_*ii*_). It is this C/A ≡ *c* > 0 that makes its appearance in all the figure captions and in Eq. 5.

The coefficients that appear in the effective elastic free-energy functional Φ(*ϵ*) (Eq. 6) conceived from renormalization of elastic stress by the active stress −*ζ*(*ρ*,*ρ*_*b*_)Δ*μ* ***I*** are given by, ${K_2} = B + \frac{{{\zeta _1}\Delta \mu {\rho _b}}}{{1 + {\zeta _2}{\rho _b}}}c\chi '({\rho _0})$, ${K_3} = - \frac{{{\zeta _1}\Delta \mu {\rho _b}}}{{1 + {\zeta _2}{\rho _b}}}{c^2}\frac{{\chi ''({\rho _0})}}{2}$ and ${K_4} = \frac{{{\zeta _1}\Delta \mu {\rho _b}}}{{1 + {\zeta _2}{\rho _b}}}{c^3}\frac{{\chi '''({\rho _0})}}{6}$. Here *ζ*_1_ < 0 and *χ*′(*ρ*_0_) > 0, so *K*_2_ goes from being positive to negative as contractility increases. The signs of the other constants are as follows: *ζ*_2_ > 0, *χ*′′(*ρ*_0_) > 0 and *χ*′′′(*ρ*_0_) < 0, so that *K*_3_ and *K*_4_ are always positive.

### Parameter values

Here we relate the values of the dimensionless parameters to real units extracted from a variety of experimental measurements. For the unit of length, $l = \sqrt {\eta /\Gamma }$ or the actin mesh size, we take 0.5*μ*m (consistent with the rough estimates in ref. ^6^) The unit of time, $k_b^{ - 1}$, can be estimated from the myosin FRAP data^8^, we find *k*_*u*_ = 0.2 ± 0.08 s^−1^, and taking the ratio *k* = *k*_*u*_/*k*_*b*_ to be 1.0, we obtain a binding rate, *k*_*b*_ = 0.2 s^−1^. The viscosity of the mesh is taken to be 50 Pa s^36^.

We can now convert all the dimensionless values into real values, and check for consistency with other experimental estimates. Thus, a dimensionless value of the bulk modulus *B* = 5 translates to *B* = 42 Pa (consistent with what can be estimated from ref. ^38^). Similarly, a dimensionless value of the magnitude of the active stress, |*ζ*_1_Δ*μ*| = 5 translates to |*ζ*_1_Δ*μρ*_*b*0_| = 42 Pa (roughly the order of magnitude estimated from ref. ^38^). Finally, the dimensionless diffusion coefficient *D* = 0.25 implies a real value of *D* = 0.01 μm^2^/s.

This implies that a dimensionless front velocity *v* = 1 (Fig. 9c) translates to a real velocity of *v* = 0.08 μm/s (consistent with the flow velocity of actomyosin reported in ref. ^6^, also see Supplementary Fig.8). Likewise, the time period of oscillation obtained in the section on “Leading order nonlinearities”, *T* = 2*π*(*η*/*k*_*u*0_(*B* + *ζ*_1_Δ*μρ*_*b*0_))^1/2^ translates to be ~20 s and the active propulsion force *f*_act_ is estimated at 30–60 pN (consistent with ref. ^38^).

### Numerical methods

Since the dynamical equations have nonlinear advection and diffusion, care must be taken in evaluating the flux due to dissipative and dispersive errors arising from spatial discretization. We use the finite volume method for spatial discretization^39^, which has been found to be useful for nonlinear advection equations^40^.

We calculate the numerical flux using the Van-Leer’s flux limiter, which uses a different formula to calculate the spatial derivative depending on how sharply the *ρ*_*b*_ profile changes in space. When the profile changes very fast, the scheme implements the upwind method^41^, which reduces the dispersion error through numerical diffusion. When the profile changes smoothly the scheme implements a second order accurate method called Lax-Wendroff method^42^. In our numerical scheme, the density (*ρ*_*b*_ or *ρ*) flux on the interface between *i* ^th^ and (*i* − 1)^th^ node is computed as,

10 $$\begin{array}{*{20}{l}}\\ {f_{i - \frac{1}{2}}^{n + \frac{1}{2}} = \frac{1}{2}{v_{i - \frac{1}{2}}}\left[ {(1 + {\theta _{i - \frac{1}{2}}})\rho _{i - 1}^n + \left( {1 - {\theta _{i - \frac{1}{2}}}} \right)\rho _i^n} \right]} \hfill \\ \\ { + \frac{1}{2}\big\vert{v_{i - \frac{1}{2}}}\big\vert \left( {1 - \Bigg\vert\frac{{{v_{i - \frac{1}{2}}}\Delta t}}{{\Delta x}}\Bigg\vert} \right)\phi _{i - \frac{1}{2}}^n\left( {r_{i - \frac{1}{2}}^n} \right)\left( {\rho _i^n - \rho _{i - 1}^n} \right)} \hfill \\ \end{array}.$$

Here, $${v_{i - \frac{1}{2}}} = \frac{{{v_i} - {v_{i - 1}}}}{2}$$ and

11 $${\theta _{i - \frac{1}{2}}} = \left( {\begin{array}{*{20}{c}}\\ { + 1,} & {{\rm{if}}{v_{i - \frac{1}{2}}}  >0} \\ \\ { - 1,} & {{\rm{if}}{v_{i - \frac{1}{2}}} \le 0} \\ \end{array}} \right.$$

12 $$r_{i - \frac{1}{2}}^n = \left( {\begin{array}{*{20}{c}}\\ {\frac{{\rho _{i - 1}^n - \rho _{i - 2}^n}}{{\rho _i^n - \rho _{i - 1}^n}},} & {{\rm{if}}{v_{i - \frac{1}{2}}}  >0} \\ \\ {\frac{{\rho _{i + 1}^n - \rho _i^n}}{{\rho _i^n - \rho _{i - 1}^n}},} & {{\rm{if}}{v_{i - \frac{1}{2}}} \le 0} \\ \end{array}} \right..$$

The function *ϕ*(*r*) is the Van-Leer flux limiter

13 $$\phi \left( r \right) = \frac{{r + |r|}}{{1 + |r|}}{\kern 1pt} .$$

The time integration is done with a total variation diminishing (TVD) 3rd order Runge–Kutta method^43^. All other derivative terms were discretized using a simple finite difference. The initial conditions were chosen from a uniform random distribution of fixed width about a uniform, unstrained configuration. We used periodic boundary conditions throughout and a time-space discretization, Δ*t* = 10^−4^ and Δ*x* = 5 × 10^−2^.

### Experimental methods and data analysis

For fluorescence time-lapse imaging, embryos at stage 7 were dechorionated with 100% bleach and mounted on No. 1 coverslip with halocarbon oil^44^. A 100×, 1.4 N.A oil immersion objective with Nikon spinning disc Eclipse Ti inverted microscope was used^44^. The system acquires images using the MetaMorph software. Starting from the most apical plane, 4–7 *z*-sections 0.5 μm apart were acquired every 1.5–4 s (depending on the experiment) using a single camera. Sum-intensity *z*-projection of slices was used for all quantifications, followed by a background subtraction using the available plugin in Fiji.

All the data analysis are done using customized code written in MATLAB. For realization of pulse shape, velocity and skewness pulses were projected on the line of their movement (for details see Supplementary Fig. 6).

### Data availability

All the relevant data are available from the authors upon request.

## Electronic supplementary material

Supplementary Information

Supplementary Files

Supplementary Movie 1

Supplementary Movie 2

Supplementary Movie 3

Supplementary Movie 4
