# Supplementary material for: Actomyosin pulsation and flows in an active elastomer with turnover and network remodeling
Source: Nat Commun. 2017 Oct 24;8:1121. doi: 10.1038/s41467-017-01130-1 (PMC5783953; doi:10.1038/s41467-017-01130-1)
Supplement: Supplementary file 1 — Supplementary Information [file 41467_2017_1130_MOESM1_ESM.pdf]

## Supplementary Note 1.

# Hydrodynamic equations of an Active Affine Elastomer

The hydrodynamic variables describing an active elastomer embedded in a fluid solvent are - (i) density of filamentous mesh, $\rho$; (ii) displacement field of filamentous mesh, $\boldsymbol{u}$; (iii) density of bound myosin minifilaments, $\rho_b$, and (iv) fluid (solvent) velocity $\boldsymbol{v}$. The linearized elastic strain is defined as $\epsilon_{ij} = 1/2(\partial_i u_j + \partial_j u_i)$. Our treatment closely follows [1, 2].

The hydrodynamic equations of the active elastomer mesh in bulk are given by,

$$\rho\ddot{\boldsymbol{u}} + \Gamma(\dot{\boldsymbol{u}} - \boldsymbol{v}) = \boldsymbol{\nabla}\cdot(\boldsymbol{\sigma}^e + \boldsymbol{\sigma}^a + \boldsymbol{\sigma}^d) \tag{1}$$

$$\dot{\rho}_b + \boldsymbol{\nabla}\cdot(\rho_b\dot{\boldsymbol{u}}) = D\boldsymbol{\nabla}^2\rho_b + \mathcal{S}_m \tag{2}$$

$$\dot{\rho} + \boldsymbol{\nabla}\cdot(\rho\dot{\boldsymbol{u}}) = \mathcal{M}\nabla^2\frac{\delta F}{\delta\rho} + \mathcal{S}_a \tag{3}$$

where $\boldsymbol{v}$ is the fluid velocity and the rest of the parameters have been defined in the main text. The friction $\Gamma$ between the mesh and cytosol can in principle depend on the mesh density. The dynamics of both actin filaments and myosin minifilaments show a turnover over a scale of minutes, which we refer to as $\mathcal{S}_a$ and $\mathcal{S}_m$, respectively.

The constitutive relations for the elastic ($\sigma^e$) and dissipative ($\sigma^d$) stresses are,

$$\sigma^e_{ij} = \left(\lambda + \frac{2\nu}{3}\right)\delta_{ij}\boldsymbol{\nabla}\cdot\boldsymbol{u} + 2\nu\left(\epsilon_{ij} - \frac{1}{3}\delta_{ij}\boldsymbol{\nabla}\cdot\boldsymbol{u}\right) \tag{4}$$

$$\sigma^d_{ij} = \eta_b\delta_{ij}\boldsymbol{\nabla}\cdot\dot{\boldsymbol{u}} + 2\eta_s\left(\dot{\epsilon}_{ij} - \frac{1}{3}\delta_{ij}\boldsymbol{\nabla}\cdot\dot{\boldsymbol{u}}\right) \tag{5}$$

Here $\lambda$, $\nu$ are the Lamé coefficients of the elastic mesh and $\eta_b$, $\eta_s$ are the bulk and shear viscosities of the mesh, respectively. The form of the active stress $\sigma^a$ is described in the main text.

The fluid hydrodynamics is given by,

$$\rho_f(\dot{\boldsymbol{v}} + \boldsymbol{v}\cdot\boldsymbol{\nabla}\boldsymbol{v}) = \eta^s_f\boldsymbol{\nabla}^2\boldsymbol{v} + (\frac{\eta^s_f}{3} + \eta^b_f)\boldsymbol{\nabla}(\boldsymbol{\nabla}\cdot\boldsymbol{v}) - \boldsymbol{\nabla}P + \Gamma(\dot{\boldsymbol{u}} - \boldsymbol{v}) \tag{6}$$

where $\rho_f$ is the density of the fluid and $\eta^s_f$ and $\eta^b_f$ are the shear and bulk viscosities of the fluid.

Finally, the pressure $P$ is eliminated by demanding total incompressibility,

$$\boldsymbol{\nabla}\cdot((1-\phi)\boldsymbol{v} + \phi\dot{\boldsymbol{u}}) = 0 \tag{7}$$

where $\phi$ denotes the volume fraction of the actomyosin mesh. For a gel, we expect $\phi \ll 1$, yielding, $\boldsymbol{\nabla}\cdot\boldsymbol{v} = 0$.

These are the complete set of equations, which we display here for completeness.

Since the cytosol is a low Reynolds number fluid, Eq. (6) reduces to a force balance condition, where the right hand side of the equation equals zero. In the main manuscript, we study the overdamped or Rouse limit where we ignore the hydrodynamics of the fluid $\boldsymbol{v}$; this is suggested by our experiments that show the actin mesh moves with respect to the fluid, and does not carry (advect) the fluid and other soluble molecules along with it, except for those which are bound to the mesh [3, 4].

Since we are interested in the over-damped dynamics of the mesh, we ignore inertia and drop the $\ddot{\boldsymbol{u}}$ term in Eq. (1). This leads to Eqs. (1), (2) and (3) in the main text.

## Supplementary Note 2. Linear Stability Analysis

For the linear analysis, we only consider Eqs. (1) and (2), with the mesh density slaved to the elastic compression $\epsilon_{ii}$ (as described in the main text). Equations (1) and (2), after going to the Rouse and overdamped limit, look like

$$\Gamma\dot{\boldsymbol{u}} = \boldsymbol{\nabla}\cdot(\boldsymbol{\sigma}^e + \boldsymbol{\sigma}^a + \boldsymbol{\sigma}^d) \tag{8}$$

and

$$\dot{\rho}_b + \boldsymbol{\nabla}\cdot(\rho_b\dot{\boldsymbol{u}}) = D\boldsymbol{\nabla}^2\rho_b - k_{u0}e^{\alpha\boldsymbol{\nabla}\cdot\boldsymbol{u}}\rho_b + k_b\rho \tag{9}$$

where we have unpacked the myosin turnover $\mathcal{S}_m = -k_u(\epsilon)\rho_b + k_b\rho$. For this linear analysis, we take the $\boldsymbol{\sigma}^a = -\zeta_1(1+\zeta'\rho)\Delta\mu\rho_b$, $\boldsymbol{\sigma}^e = B\boldsymbol{\nabla}\cdot\boldsymbol{u}$ and $\boldsymbol{\sigma}^d = \eta\boldsymbol{\nabla}\cdot\dot{\boldsymbol{u}}$. Since $\boldsymbol{\sigma}^a$ is contractile, we take $\zeta_1 < 0$. In this one-constants approximation, $B$ and $\eta$ are given by $\lambda + 2\nu$ and $\eta_b + \frac{4}{3}\eta_s$, respectively. For convenience, we set $\zeta' = 1$.

These equations can be rewritten in dimensionless form with time ($t$) and space ($\boldsymbol{x}$) in units of $k_b^{-1}$ and $l = \sqrt{\frac{\eta}{\Gamma}}$, respectively, leading to the redefinitions,

$$\begin{aligned} \frac{u}{l} &\to u \\ \rho_b/\rho_{b0} &\to \rho_b \\ \frac{B}{\Gamma k_b l^2} &\to B \\ \frac{\zeta_1 \Delta\mu \rho_{b0}}{\Gamma k_b l^2} &\to \zeta_1 \Delta\mu \\ \frac{k_{u0}}{k_b} &\to k \\ \frac{D}{k_b l^2} &\to D \end{aligned} \tag{10}$$

and the following equations in dimensionless form

$$(1 - \nabla^2)\dot{\boldsymbol{u}} = (B + \zeta_1 \Delta\mu)\nabla^2 \boldsymbol{u} - \zeta_1 \Delta\mu \boldsymbol{\nabla} \rho_b$$

and

$$\dot{\rho}_b + \boldsymbol{\nabla} \cdot (\rho_b \dot{\boldsymbol{u}}) = D\boldsymbol{\nabla}^2 \rho_b - k e^{\alpha \boldsymbol{\nabla} \cdot \boldsymbol{u}} \rho_b + \rho \ .$$

Upon linearizing about the homogeneous, unstrained fixed point $(u_0, \rho_{b0}, \rho_0)$, we obtain,

$$(1 - \nabla^2)\delta\dot{\boldsymbol{u}} = (B + \zeta_1 \Delta\mu)\nabla^2 \delta\boldsymbol{u} - \zeta_1 \Delta\mu \nabla \delta\rho_b$$

and

$$\delta\dot{\rho_b} + \nabla \cdot \delta\dot{\boldsymbol{u}} = D\nabla^2 \delta\rho_b - k(\alpha + c)\boldsymbol{\nabla} \cdot \delta\boldsymbol{u} - k\delta\rho_b$$

where we have used the fact that the fluctuation in $\rho$ is slaved to the compression, $\delta\rho = -c\epsilon_{ii}$ ($c > 0$).

On Fourier transforming the above equations in space, $f(\boldsymbol{q}, t) = \int_{-\infty}^{\infty} f(\boldsymbol{x}, t) e^{-i\boldsymbol{q}\cdot\boldsymbol{x}} d\boldsymbol{x}$, we obtain the matrix equation,

$$\begin{bmatrix} \delta\dot{u} \\ \delta\dot{\rho_b} \end{bmatrix} = \begin{bmatrix} -\left(\frac{q^2}{1+q^2}\right)(B + \zeta_1 \Delta\mu) & -\left(\frac{iq}{1+q^2}\right)\zeta_1 \Delta\mu \\ iq\left(\frac{q^2}{1+q^2}(B + \zeta_1 \Delta\mu) - (\alpha + c)k\right) & -\left(k + \frac{q^2}{1+q^2}\zeta_1 \Delta\mu + q^2 D\right) \end{bmatrix} \times \begin{bmatrix} \delta u \\ \delta\rho_b \end{bmatrix} \tag{11}$$

Solving (11) for the two eigenvalues, $\lambda_+$ and $\lambda_-$, we obain the general solution,

$$u(\boldsymbol{q}, t) = u_1(\boldsymbol{q}) e^{\lambda_+ t} + u_2(\boldsymbol{q}) e^{\lambda_- t} \tag{12}$$

where, $\lambda_\pm = \lambda_1 \pm \sqrt{\lambda_2}$, with

$$\lambda_1 = \frac{-\left(k(1 + q^2) + Dq^2(1 + q^2) + q^2(B + 2\zeta_1 \Delta\mu)\right)}{2(1 + q^2)}$$

$$\lambda_2 = \frac{\left(k\left(1 + q^2\right) + q^2\left((B + D + Dq^2\right) + 2q^2 \zeta_1 \Delta\mu\right)^2 - 4q^2\left(1 + q^2\right)\left(B\left(k + Dq^2\right) + \left(Dq^2 + k(1 + c + \alpha)\right)\zeta_1 \Delta\mu\right)}{4(1 + q^2)^2}$$

From the dispersion relations, we obtain 4 phases (a) stable ($Im[\lambda_\pm] = 0$), (b) damped oscillations, (c) unstable oscillations, and (d) contractile instability ($Im[\lambda_\pm] = 0$), corresponding to the dispersion curves shown in Fig. S2. The phase boundary in Fig. 1b of the main text is obtained by specifying a particular value of $q$ (we have taken $q = 2$), or by restricting $q$ to lie between $[q_{min}, q_{max}]$, where $q_{min} = 2\pi/L$ and $q_{max} = 2\pi/l$ ($= 2\pi$, in rescaled units).

The phase boundary between the *stable and damped traveling wave phases* can be obtained from the solution of $Im\,[\lambda_\pm] = 0$,

$$\zeta_1 \Delta\mu\Big|_{S_d \to S_t} = \frac{1}{2}\left(-B - \sqrt{2Bk - k^2 + 2BDq^2 - 2Dkq^2 - D^2 q^4}\right)$$

The *stable phase* crosses over to the *unstable phase*, when

$$-\left(B + 2\zeta_1 \Delta\mu + k + Dq_c^2\right) \geq 0 \tag{13}$$

(obtained from $Re\,[\lambda_\pm] = 0$), where $q_c$, is the fastest growing mode,

$$q_c = \left(\sqrt{-\frac{B + 2\zeta_1 \Delta\mu}{D}} - 1\right)^{\frac{1}{2}} . \tag{14}$$

The phase boundary between the *unstable oscillatory and contractile instability phases* can be obtained from the solution of $Im\,[\lambda_\pm] = 0$,

$$\zeta_1 \Delta\mu\Big|_{O\to CI} = \frac{1}{2}\left(-B + \sqrt{2Bk - k^2 + 2BDq^2 - 2Dkq^2 - D^2q^4}\right)$$

Precisely at the stable-unstable phase boundary, since $Re\,[\lambda_\pm] = 0$, the solutions correspond to left and right traveling waves for $\rho_b$ (and $u$), of the form

$$\rho_b(x,t) = A\cos(q_c\, x \pm w_c\, t + \theta) \tag{15}$$

where the wavelength $q_c$ and frequency $w_c$ of the wave are given by, $q_c^* = \left(\frac{k}{D}\right)^{\frac{1}{4}}$ and $\omega_c^* = \sqrt{\frac{kB}{2}}$, leading to a wave-speed, $v_c^* \equiv \frac{\omega_c^*}{q_c^*} = \left(\frac{kDB^2}{4}\right)^{\frac{1}{4}}$.

## Supplementary Note 3. **Strain dependent unbinding**

For the turnover of bound myosin filament density, we allow for a possible strain-induced unbinding of the Hill-form, $k_u = k_{u0} e^{\alpha \nabla \cdot \mathbf{u}}$. The sign of the coefficient $\alpha$ can be taken to be either positive or negative (Fig. S3a) : $\alpha > 0$ implies a local extension (compression) of the mesh will increase (decrease) the myosin unbinding, while $\alpha < 0$ implies a local compression (extension) of the mesh will increase (decrease) the myosin unbinding. The choice $\alpha = 0$ implies that the myosin unbinding rate is a constant, independent of mesh deformation. We thus cover all possibilities.

Changing the sign of $\alpha$ only affects the placement of the phase boundaries, but not the qualitative aspects of the phases, see Fig. S3b,c. We find that the oscillatory phase exists, as long as the eigenvalue $Im[\lambda_{\pm}] < 0$, from which get the maximal (positive) value $\alpha_{max}(B, \zeta_1, \ldots)$, beyond which there are no oscillations. Thus to get oscillations for a given set of (other) parameters, we have to set $-\infty < \alpha < \alpha_{max}$. We have taken $\alpha > 0$ (but smaller than $\alpha_{max}$) in all numerical results presented in the main text.

# Supplementary Figures

## 1. Germband Tissue

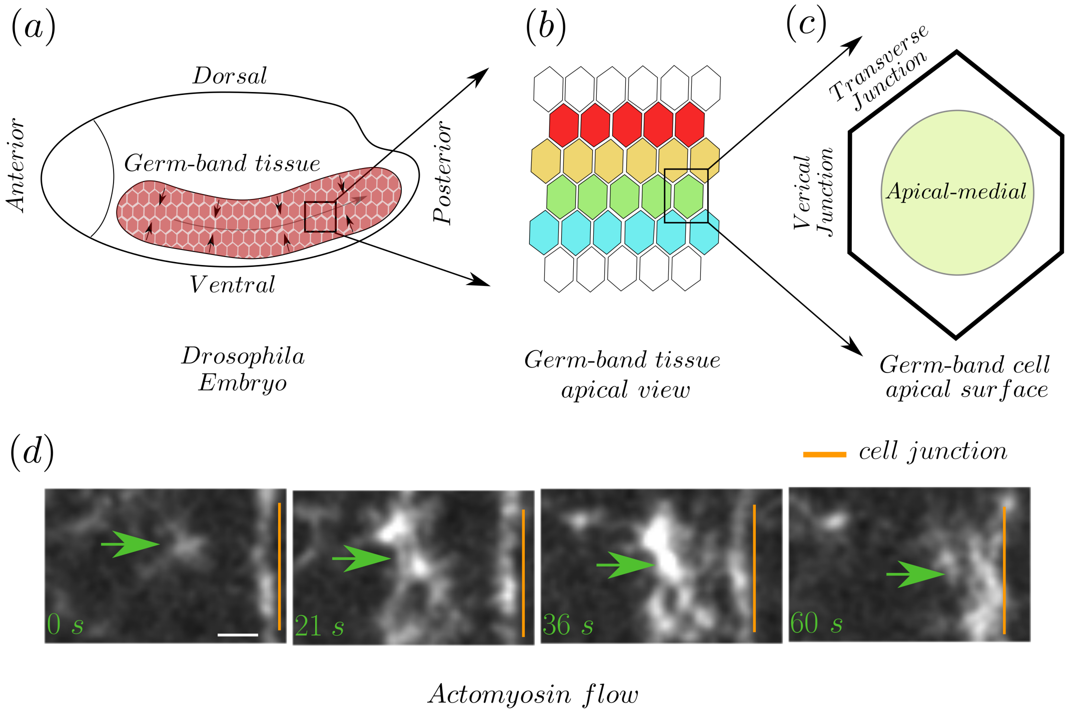


Supplementary Figure 1: Germband tissue and cells in a drosophila embryo. (a) The germband tissue changes shape (extends along anterior-posterior (AP) axis and contracts along dorsal-ventral (DV) axis) as indicated by the arrows. (b) Cells in germband layer go through $T_1$-transitions where the vertical junctions of the cell get contracted along DV and then get elongated along AP. These changes in the cells are driven by cortical actomyosin activity, pulsation and flow. (d) MRLC-GFP tagged myosin II intensity plots show pulsation and flow in apical plane of one germband cell. MRLC stands for myosin regulatory light chain. The scale bar in the leftmost panel of (d) is 1 $\mu m$.

## 2. Dispersion relations and phase diagram

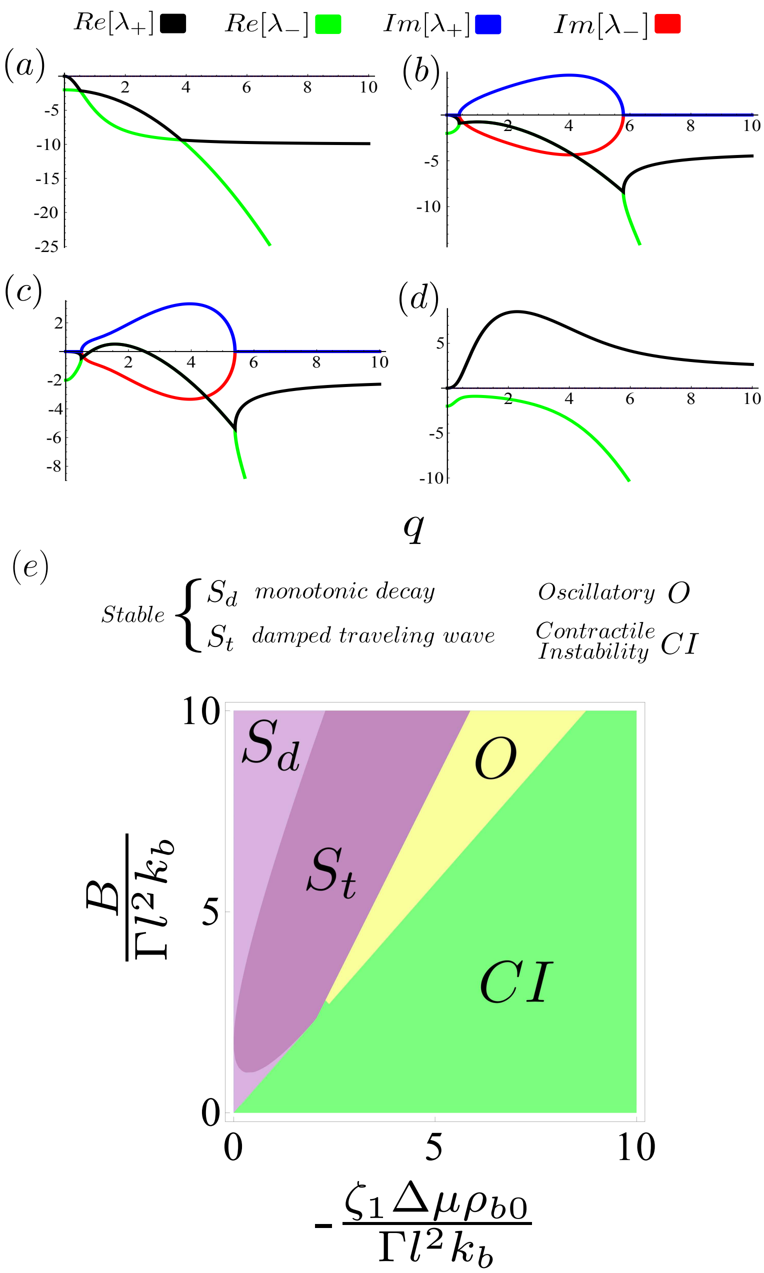


Supplementary Figure 2: Dispersion relations from linear stability:(a-d) Typical dispersion curves obtained from linear stability analysis, showing the complex roots $\lambda_\pm$ as a function of wave-vector $q$. Colour code displayed above. Panels shows typical behaviour in the (a) stable, (b) damped oscillations, (c) unstable oscillations, and (d) contractile instability phases. These phases correspond to the phase diagram below. (e) Linear stability phase diagrams in effective elastic stress density vs. contractile stress density at $k = 1.0$. Rest of the parameters are $\alpha = 0.1, c = 0.1, D = 0.1$.

## 3. Strain dependent unbinding

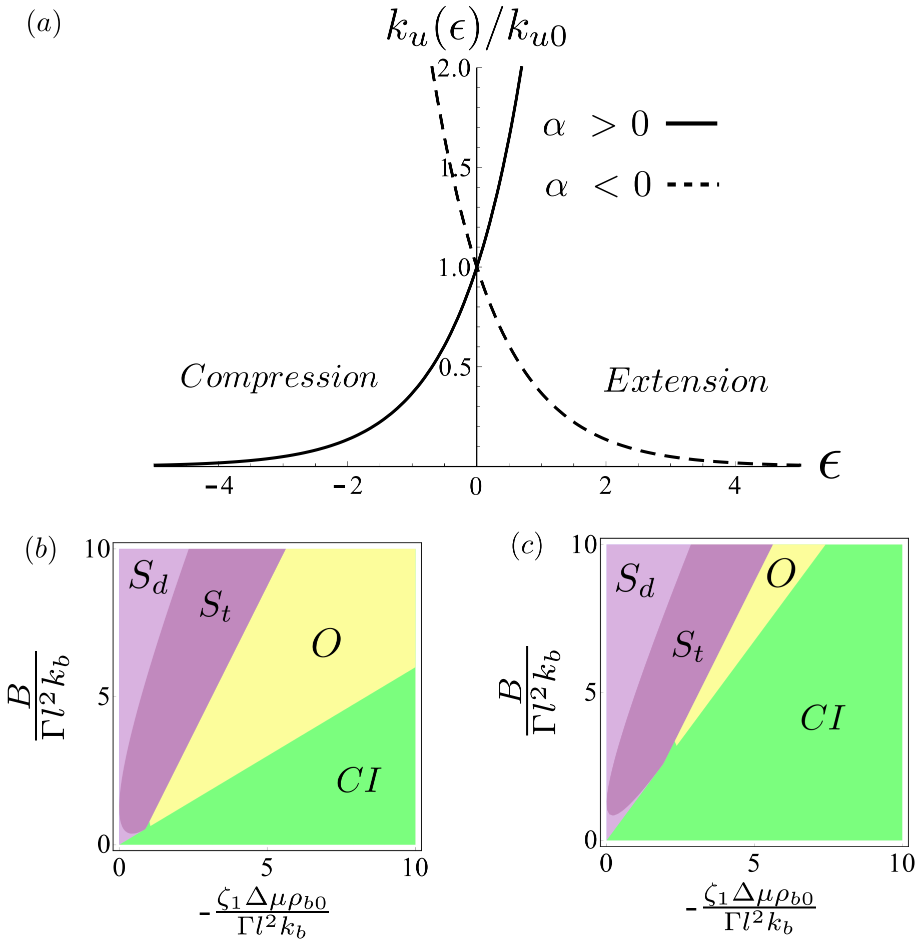


Supplementary Figure 3: (a) Plot of unbinding rate $k_u(\epsilon)/k_{u0}$ with strain $\epsilon$, for both positive and negative $\alpha$. (b,c) Sign of $\alpha$ does not change the qualitative nature of the phase diagram in the effective elastic stress density versus active stress density (nomalized to the frictional stress), as long as $\alpha < \alpha_{max}$. The color scheme and legend are as in Supplementary Fig.2, (b) $\alpha = -0.5$ and (c) $\alpha = 0.5$. Other parameters are $k = 10$, $c = 0.1$ and $D = 0.1$. Note that the regime of oscillations is larger when $\alpha < 0$.

## 4. Effective free energy functional

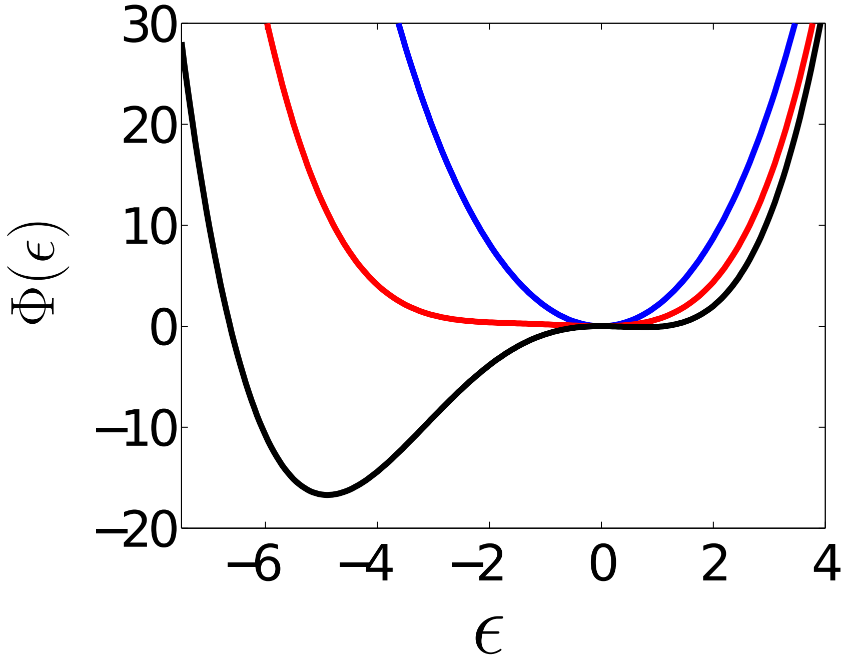


Supplementary Figure 4: Effective elastic free energy $\Phi(\epsilon)$ as a function of strain for three different values of active stress. At low active stress, $\Phi$ has a single minimum at $\epsilon = 0$, the free-energy profile has a lower curvature (corresponding to lower renormalized elastic modulus) than the passive elastomer. At intermediate values of active stress, there appears a second minimum at $\epsilon = \epsilon_0$. At higher values of active stress, the minimum at $\epsilon = 0$ becomes unstable.

## 5. Timescales of oscillation, front movement and contractile collapse

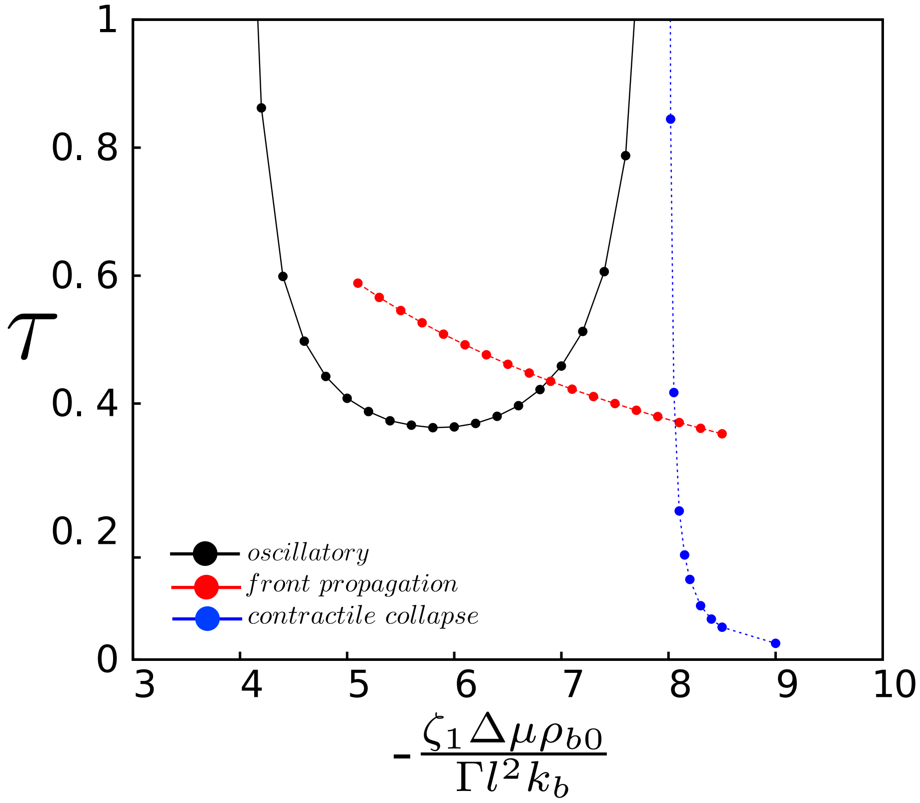


Supplementary Figure 5: Time scale of various events - oscillation, front propagation and contractile collapse - as a function of the active stress, obtained from theory, shows that with increasing active stress one first encounters the oscillatory phase, then the pulse propagation and finally the collapse. The boundaries of these transitions support coexisting behaviours. The oscillatory and contractile collapse timescale values are calculated from the one mode analysis with the rest of the parameters fixed at $B = 8$, $\alpha = 1$, $c = 0.1$ and $k = 5$.

## 6. Protocol for Image Analysis

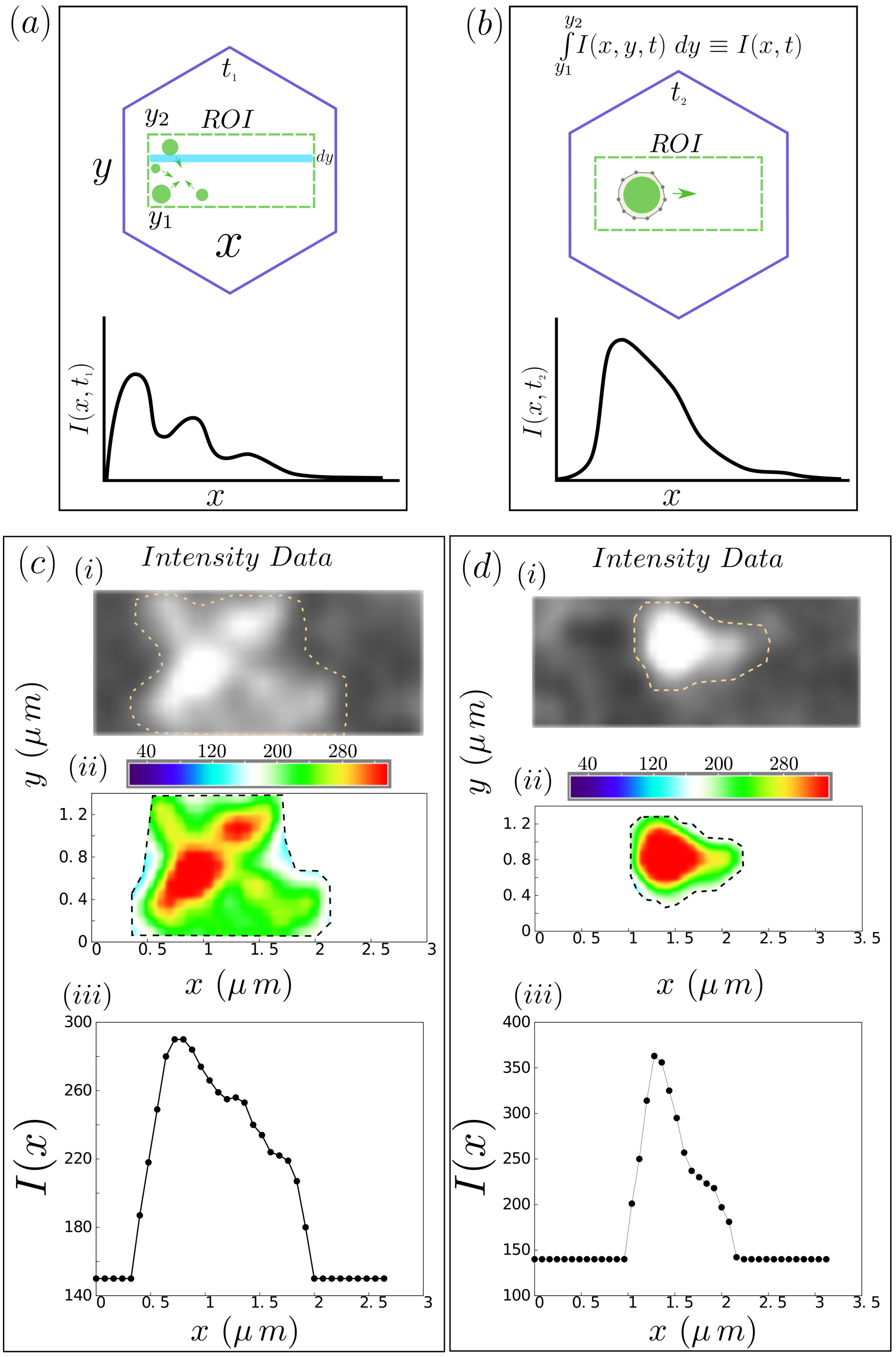


Supplementary Figure 6: Protocol for image analysis of the experiments on the dynamics of labeled medial myosin. (a,b) We collect the 2d intensity map ($I(x, y, t)$) of myosin (green dots) at different times, e.g., $t_1, t_2$ (with $t_2 > t_1$), within a thin rectangular strip (green rectangle) chosen so that it is not contaminated by signals at the cell boundary. After background subtraction, we integrate the intensity $I(x, y, t)$ along $y$ between the limits $y_{min}$ and $y_{max}$, along each thin rectangular strip (blue). This gives us the 1-dimensional profile $I(x, t)$ vs. $x$, as shown schematically here. (c,d)(i) We carry out the above protocol for the labeled myosin-dense images obtained in two different experiments. (ii) After background subtracting the intensity maps, we plot $I(x, y, t)$ in the $x - y$ plane. (iii) The 1D projection $I(x, t)$ is plotted versus $x$.

## 7. Coalescence Events

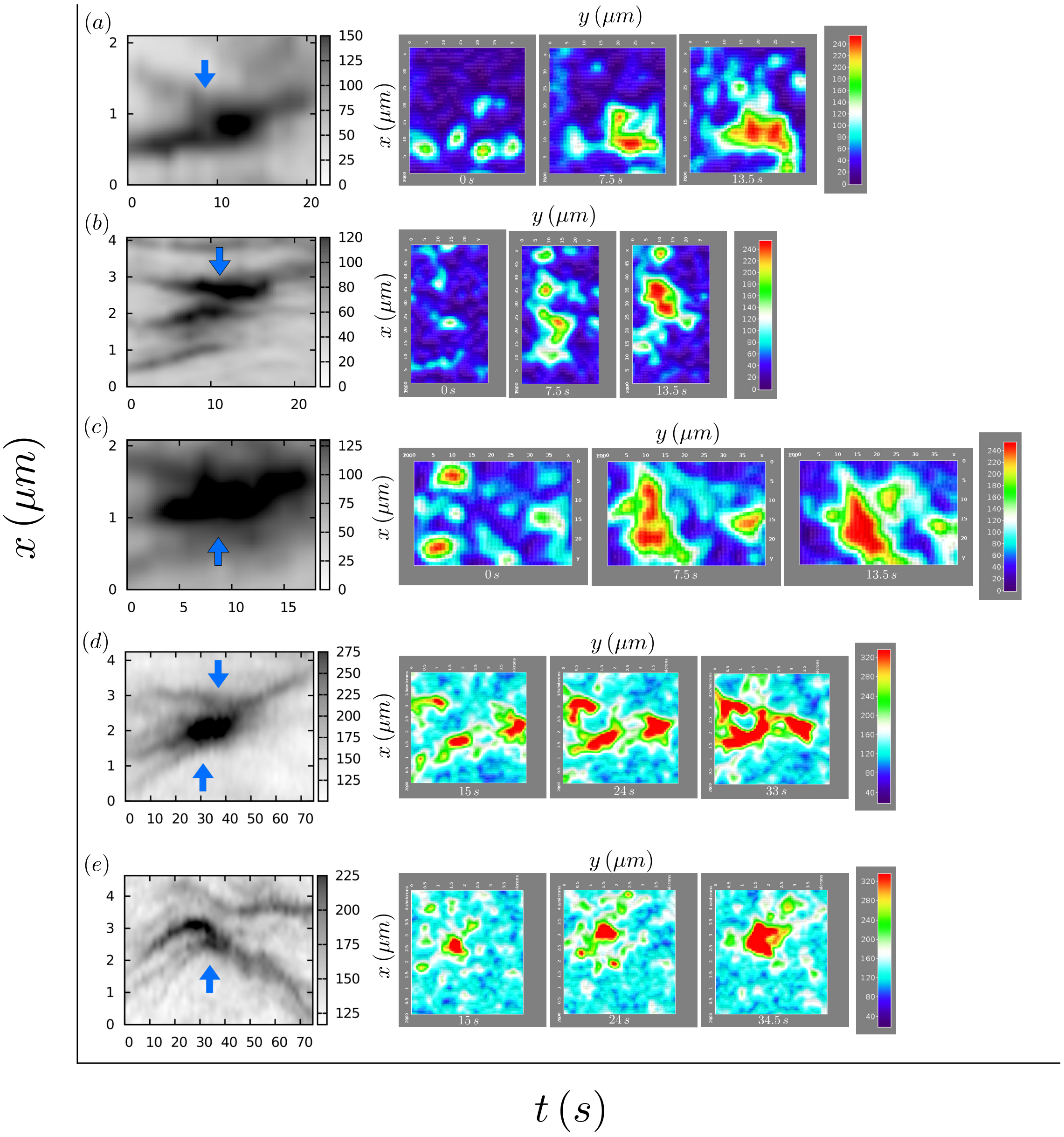


Supplementary Figure 7: (a-e) Kymographs of the projected myosin intensity from experimental intensity data show clear coalescence events (arrows). The corresponding 2d intensity maps at different time instants, are shown alongside each kymograph. The images and kymographs clearly show show growth and coalescence, followed by movement.

## 8. Velocity Distribution

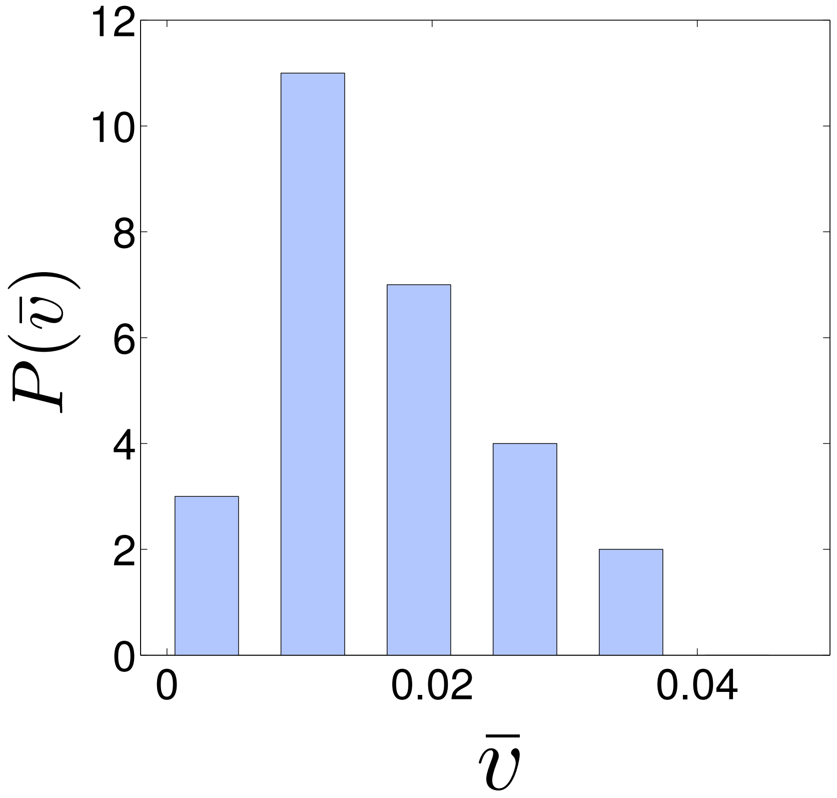


Supplementary Figure 8: Probability distribution $P(\bar{v})$ of average velocity $\bar{v}$ of moving myosin-dense clusters. For each moving cluster, we compute the average $\bar{v}$ over time before any coalescence events. The bin size is $0.008\,\mu\mathrm{m\,s}^{-1}$. Data collected from 27 myosin-dense clusters from 20 cells.

## 9. Velocity Reversals

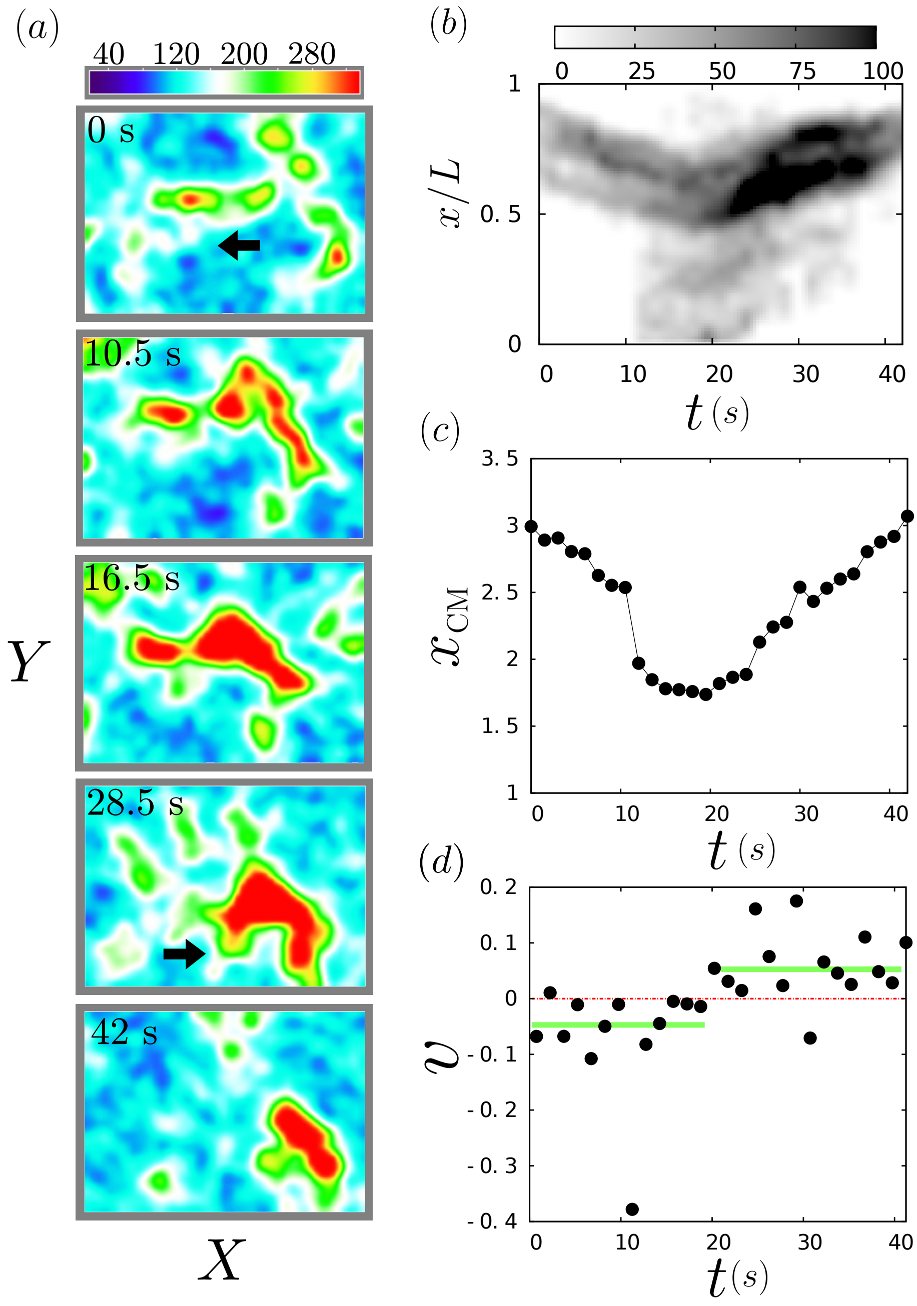


Supplementary Figure 9: Direction reversals of the moving myosin-dense cluster indicate that the driving force for flow does not come from the boundary but is intrinsic to the myosin-dense regions. (a) The 2d intensity maps at different time instants, shows a myosin-dense cluster moving towards the right, which following coalescence, then reverses its direction (movement is along the direction of arrow). (b) Corresponding kymograph clearly shows this velocity reversal. From the projected intensity maps, we quantify the velocity reversals by (c) the centre of mass of the myosin-dense cluster $x_{CM}$ versus time and (d) the moving cluster velocity versus time. There is some reorganisation of the myosin-dense cluster upon coalescence.

# 10. Comparison of affine theory with experiments

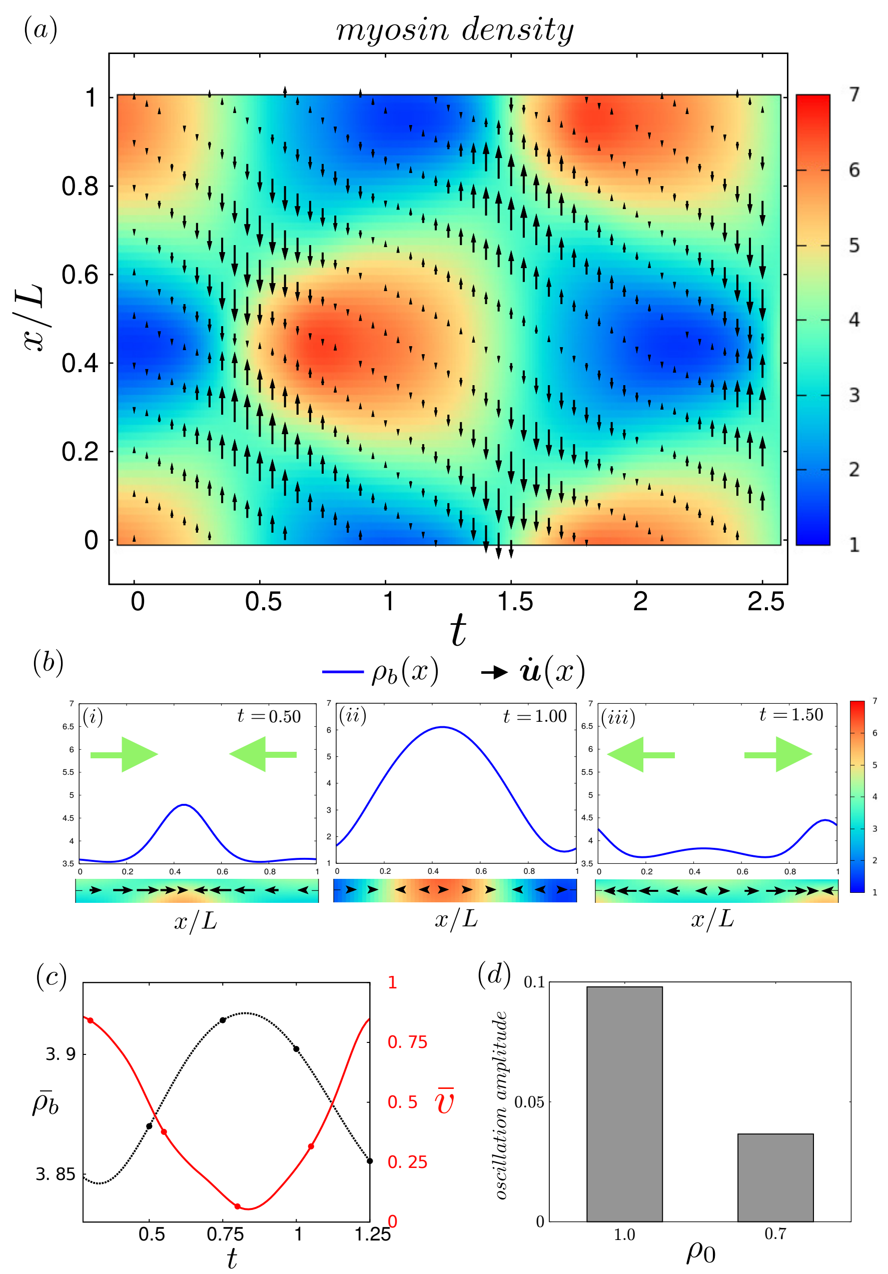


Supplementary Figure 10: We compare the characteristics of the oscillatory phase with the pulsation seen in experiments on germband cells as reported in [3] (specifically Fig. 4 of [3]). (a)-(c) shows that *advection* is crucial to obtain oscillations of bound myosin. (a) Space-time plot (kymograph) of the bound myosin density (colour bar) against a foreground of arrows indicating the local velocity vector $\dot{\boldsymbol{u}}$ (whose magnitude is given by the size of the arrows). This velocity vector describes the advection of myosin by the actin mesh, and shows that local convergence of velocity is associated with increased myosin density (and vice versa). This recapitulates the advection-myosin density profiles shown in Fig. 4a of [3]. (b) This panel shows spatial profiles extracted from the above figure at three specific time instants. There is a definite correlation between convergent velocity vectors (black arrows) and increased myosin density (blue graph) between $t = 0.5$ and $t = 1$, and divergent velocity vectors (black arrows) and reduced myosin density (blue graph) between $t = 1$ and $t = 1.5$. Green arrows show overall convergence (divergence) of advection. Our results here are consistent with Fig. 4a-c of [3]. (c) Time variation of spatially averaged myosin density $\bar{\rho}_b$ (black line) and advection speed $\bar{v}$ (obtained from magnitude of $\dot{\boldsymbol{u}}$, red line) during an oscillation cycle. The graph of $\bar{v}$ is shifted to the left with respect to the myosin density graph by an amount 0.2, indicating that the advection is a cause for local enhancement of myosin density. Our results are entirely consistent with Fig. 4m of [3]. (d) The amplitude of the oscillation decreases when we reduce actin mesh density $\rho_0$, here we compare $\rho_0 = 1$ with $\rho_0 = 0.7$. Compare this to the actin perturbation experiments, Fig. 4f of [3]. Parameter used here are, $B = 8$ , $-\zeta_1\Delta\mu = 5.2$, $k = 0.2$ , $D = 0.25$, $\alpha = 3$, $c = 0.1$.

# References


[1] S. Banerjee, T.B. Liverpool and M.C. Marchetti, Generic phases of cross-linked active gels: Relaxation, oscillation and contractility, Europhys. Lett. **96**, 58004 (2011).

[2] S. Banerjee and M.C. Marchetti, Instabilities and oscillations in isotropic active gels, Soft Matter **7**, 463 (2011).

[3] A. Munjal, J-M. Philippe, E. Munro and T. Lecuit, A self-organized biomechanical network drives shape changes during tissue morphogenesis, Nature (2015).

[4] M. Rauzi, P-F. Lenne and T. Lecuit, Planar polarized actomyosin contractile flows control epithelial junction remodelling, Nature **468**, 1110-1114 (2010).
