# Supplementary material for: Actomyosin pulsation and flows in an active elastomer with turnover and network remodeling
Source: Nat Commun. 2017 Oct 24;8:1121. doi: 10.1038/s41467-017-01130-1 (PMC5783953; doi:10.1038/s41467-017-01130-1)
Supplement: Supplementary file 2 — Supplementary Files [file 41467_2017_1130_MOESM2_ESM.pdf]

**Description of Additional Supplementary Files**

**File Name: Supplementary Movie 1**

Description: In-vivo imaging in a typical germband cell of the drosophila wing imaginal disc, shows nucleation, growth and coalescence of an actomyosin-dense cluster (GFP tagged myosin-RLC (WT)) and its subsequent flow towards a vertical cell junction.

**File Name: Supplementary Movie 2**

Description: Small myosin-dense speckles appear and disappear in the background of larger myosin-dense cluster dynamics. These small speckles have a lifetime of about 5-10 s.

**File Name: Supplementary Movie 3**

Description: Numerically calculated dynamics of the spatial profile ofSuppleme
yosin density (versus scaled coordinate x/L, where L is the cell size) starting from statistically uniform initial conditions. Note the nucleation, growth, coalescence of myosin enriched regions and subsequent formation of a spatially localized traveling front.

Parameter values are B=6, $-\xi_1\Delta\mu$=5.5, k=0.25, $\alpha$=1, D=0.25. Periodic boundary conditions.

**File Name: Supplementary Movie 4**

Description: Numerically calculated spatial profile of bound myosin density (red) and strain (blue) within the spatially localized traveling front. Myosin contractility compresses the actin mesh locally, resulting in a localized extension region ahead of the leading edge of the traveling pulse. The traveling pulse maintains its shape as it moves.

Parameter values are B=6, $-\xi_1\Delta\mu$==5.6, k=0.2, $\alpha$=1, D=0.25. Periodic boundary conditions.
